# Supplementary material for: Benchmarking integration of single-cell differential expression
Source: Nat Commun. 2023 Mar 21;14:1570. doi: 10.1038/s41467-023-37126-3 (PMC10030080; doi:10.1038/s41467-023-37126-3)
Supplement: Supplementary file 1 — Supplementary Information [file 41467_2023_37126_MOESM1_ESM.pdf]

Benchmarking integration of single-cell differential expression

Supplementary Information

Hai C. T. Nguyen[1,6], Bukyung Baik[1,6], Sora Yoon[1,5], Taesung Park[2,3], Dougu Nam[1,4]

[1]Department of Biological Sciences, Ulsan National Institute of Science and Technology, Ulsan 44919, Republic of Korea

[2]Department of Statistics, Seoul National University, Seoul 08826, Republic of Korea

[3]Interdisciplinary Program in Bioinformatics, Seoul National University, Seoul 08826, Republic of Korea

[4]Department of Mathematical Sciences, Ulsan National Institute of Science and Technology, Ulsan 44919, Republic of Korea

[5]Present address: Department of Genetics, University of Pennsylvania Perelman School of Medicine, Philadelphia, PA 19104

[6]These authors contributed equally: Hai C. T. Nguyen, Bukyung Baik

Correspondence should be addressed to D.N. (email: dougnam@unist.ac.kr).

Supplementary Notes

On our study design

We considered balanced DE analysis and assumed the cell class information was given *a priori* from clinical information. If some batches do not contain both the conditions to be compared, we have two choices: (1) remove the unbalanced batches or (2) just use naïve DE analysis for pooled uncorrected data. If large batch effects are expected and the unbalanced batches take a negligible portion, then the first choice would be reasonable. When the cell labels were not given, the cells should be clustered in the first step to classify cells where a range of feature selection, BEC and clustering algorithms were considered[1]. Some BEC methods did not yield the corrected expression data; however, they can still serve to identify the cell classes. Once the cell labels were obtained, then all the genes available were reincluded in the analysis and the optimal DE workflow was sought. In short, cell clustering and DE analysis are in general separate processes, and corresponding methods might be selected from respective benchmark studies.

Known disease genes (GDA score ≥0.5) exclusively detected by lung epithelial cell analyses

EGFR gene encodes a cell membrane receptor tyrosine kinase. It is involved in multiple signaling transduction cascades, known to play an important role in development of tumor malignancy through cell cycle progression, apoptosis inhibition, induction of angiogenesis through several downstream pathways including RAS/RAF/MAPK pathway.[2] *KRAS* is well known member of RAS gene family as an oncogene involved in RAS/RAF/MAPK signaling pathway. In general, *KRAS* is activated in response to signaling from upstream Receptor Tyrosine Kinases including EGFR.[3] CTNNB1 is a key regulator of Wnt signaling pathway and maintains cell-cell adhesion.[4] Increased *CTNNB1* expression can promote tumor invasion and metastasis by loss of epithelial structural integrity.[5] Lastly, *ERBB2* encodes another receptor tyrosine kinase and belong to same Erbb family where *EGFR* is included. Increased expression of *ERBB2* is known to trigger multiple signaling pathways such as RAS/RAF/MAPK signaling pathway, which plays an important role in regulating cell proliferation, migration, and differentiation.[2]

Implementation of benchmark methods

Here we described how each benchmark method was implemented. For all scRNA-seq data, we removed the genes with the zero expression values > 95% and the cells with high mitochondrial genes. All our codes are available at Github: https://github.com/noobCoding/Benchmarking-integration-of-scRNAseq-differential-analysis.

- *Combat*[6] (*sva* R package ver. 3.38.0): Each library of the raw count data was scaled to the median library size, and then log-scaled using log1p R function. The resulting normalized data and batch information were fed to *Combat* function. We used the default options as follows: *ref.batch=NULL* indicating *Combat* will determine the reference batch for the adjustment; *par.prior=TRUE* indicating parametric adjustments will be used;

mean.only=FALSE indicating *Combat* will correct all values in batches with the median-library adjustment. *Combat* outputs the corrected data in their log-scale.

- *MNNCorrect*[7] (*batchelor* R package ver. 1.6.3): Raw count data were log-scaled using LogNormalize function of Seurat R package. The log-normalized count data were used as input for *MNNCorrect*. *MNNCorrect* uses the first batch as the coordinating system to integrate other batches; thus, the selection of the first batch is a critical step. *MNNCorrect* also provides the option *auto.merge* to search for the optimal order for merging batches to increase the stability of the correction. We used *auto.merge=TRUE* and other default parameters including the cosine normalization for input datasets.

- *scMerge*[8] (R package ver. 1.6.0): *scMerge* requires a list of highly variable genes (HVGs) as an input; thus, we identified the HVGs of the log-normalized data. We have generated Seurat object of log-scaled raw count data, using *LogNormalize* function, and selected 1000 highly variable genes using the function *FindVariableFeatures*. We then used the function *SingleCellExperiment* to create an object of raw count data. Then, we have run logNormCounts function from scuttle R package to provide log-scaled input for *scMerge* function. Besides, *scMerge* asks for a set of 'negative control genes' [8] (e.g., stably expressed genes-SEGs) for its processing. We used the built-in function *SEGIndex* to select stably expressed genes with zero rate less than 80% (default). This threshold caused an error when analyzing very sparse data. To prevent this error, we have changed the cutoff 80% to 90% (named as *SEGIndex90*). Throughout our study, we found that the "stability threshold" of 0.5 was suitable. The supervised analysis of scMerge that incorporated the known cell group labels (*cell_type* parameter) was used.

- *limma*[9] (R package ver. 3.46.0): The raw count data were log-scaled using LogNormalize function of Seurat to be used for limma. Because of the wide range of applications of *limma*, we tested *limma* in three different ways: (1) Generation of BEC data (*limma_bec*), (2) DE analysis using limmatrend or limmavoom with or without batch covariate, and (3) DE analysis using limmatrend for BEC data obtained from *Combat*, *MNNCorrect*, or *scMerge*. For *limma_bec*, the function *removeBatchEffect* provides BEC data in their log-scale, which can be used as input for Wilcoxon test or limmatrend. The limma-based DE analysis can be performed by running *lmFit* and *eBayes* functions. *lmFit* function takes the design matrix and log-scaled count data as input, and eBayes function accepts the output of *lmFit* to finally provide DE *p*-values and logFC values for all genes. The first approach is *limma* with *voom* transformation which takes raw count data. Library sizes were multiplied by the normalization factors obtained using *calcNormFactors* function of *edgeR* and fed to the parameter *lib.size* of *voom.* Output of *voom* (log-scaled data and precision weight) is then passed to *lmFit* function. For *limmatrend* approach, the raw count data were log-scaled using *cpm* function of edgeR package with the option *log=T*. Limmatrend was implemented by setting the parameter *trend=TRUE* of the function *eBayes.* Lastly, we applied *limmatrend* to BEC data. Because

all the BEC data were in their log-scale, they were directly used for *lmFit* function. Additionally, batch covariate was incorporated in the model by altering the design matrix of *lmFit*.

- *Seurat*[10] (R package ver. 4.0.2): We normalized each batch using function *SCTransform* incorporating the 'batch' information using *batch_var* parameter. The raw count data were log-scaled using run LogNormalize function and corresponding Seurat object was used as input for *SCTransform.* We have set the parameters *variable.features.n* and *ncells* to the numbers of total genes and cells, respectively. Additionally, the option *return.only.var.genes = F* was set to obtain the normalized data with the same numbers of gene and cell as those of original data. The normalized data are stored in the data slot of SCT assay.We can obtain the batch-effects corrected values from the transformed data that are used for both sign preservation tests (Seurat_BEC) and DE test (Seurat_Wilcox).

- *Wilcoxson* (*ranksum*) *test*[11] : This test is used to compare distributions of two sample groups based on the ranks rather than the expression values. This test was implemented using *FindMarkers* function in *Seurat* package with the following parameters: *logfc.threshold=0*, *min.cells.feature=0*, *min.cells.group=0*, *min.pct=0*, *only.pos=FALSE* to obtain the full list of ranked genes (whether significant or not). *FindMarkers* function was applied to Seurat object. When *Wilcoxson test* is applied to BEC data, we have generated Seurat object of batch corrected data in their log-scale and run *FindMarkers* function directly. In case of Raw_Wilcox, we have generated Seurat object of raw count data and run LogNormalize function followed by scale function before running *FindMarkers* function.

- *MAST* [12] (R package ver. 1.16.0): This test is implemented using *FindMarkers* function in *Seurat* package like *Wilcoxson test.* We used the option *test.use='MAST'* to run *MAST,* other parameters set as follows: *logfc.threshold=0*, *min.cells.feature=0*, *min.cells.group=0*, *min.pct=0*, *only.pos=FALSE* to obtain the full list of ranked genes (whether significant or not). The raw count was taken into Seurat object and log-scaled using LogNormalize function before running *FindMarkers* function. We used the default settings for all datasets except incorporating the batch covariate using *latent.vars* parameter of *FindMarkers*.

- *DESeq2* [13] (R package ver. 1.30.1): We added pseudocount 1 to all data entries to prevent the situation that each gene includes at least one 0, which leads to an unsolvable problem for the function. Or, alternately, using the raw count with *sfType='poscount'* can also do the trick. However, we found that the first option usually gave the better performance. Count matrix with the pseudocount, the cell labels and the contrasting design formula are then fed as input for the function *DESeqDataSetFromMatrix* to create so-called *DESeq2* object which was used for the input of DESeq2 function. The output is passed to the function *lfcShrink* for estimating the shrinkage of effect size. As suggested by the authors of this method, we specified to use the *apeglm* method for effect size shrinkage [14], which often improved the performance. By customizing the design formula, we can incorporate the batch covariate information. When involved with *ZINB-WaVE* method, DESeq2 takes *observationalWeights* in addition

to the same design formula and raw count data with pseudocount 1. Detailed description is in *ZINB-WaVE* section. When used for meta-analysis, DESeq2 was run on each batch/dataset separately and the individual outputs for each batch/dataset were combined.

- *edgeR*[15] (R package ver. 3.32.1): The raw count data were fed to *DGEList* object along with the corresponding group information as meta data. The *edgeR*'s built-in normalization was applied via the function *calcNormFactors* tha implemented trimmed mean of M-values (TMM) normalization by default. Then, we estimated the dispersion parameter for the negative binomial model by feeding the normalized data and the modeled design to the function *glmQLFit*, *which* provided DE analysis results such as *p*-values and logFC values as output. Beside testing the batch covariate, we also tested *edgeR_DetRate* [16] version which used the fraction of detected genes per cell as another covariate. When involved with *ZINB-WaVE*, edgeR takes *observationalWeights* in addition to the same design formula and raw count data. Detailed description is in the *ZINB-WaVE* section. When used for meta-analysis, edgeR was run on each batch/dataset separately and the individual outputs for each batch/dataset were combined.

- *ZINB-WaVE*[17] (R package ver. 1.12.0): We used the function *SingleCellExperiment* to create an object from the raw counts data and the corresponding meta information. The *zinbwave* function is applied to the object to provide the low-dimensional representation of the data from the perspective of ZINB-WaVE. (https://bioconductor.org/packages/release/bioc/vignettes/zinbwave/inst/doc/intro.html#differential-expression). There are two ways for DE analysis using ZINB-WaVE output

- To obtain the BEC data, the parameter *normalizedValues* should be set as "TRUE" to let the function return the corrected data, denoted as ZW_BEC data. These corrected data were used for *Wilcoxson test.*
- To use the observation weights, the parameter *observationalWeights* of the function *zinbwave* is required to be "TRUE". These weights are used as input in addition to the raw count data for DESeq2 or edgeR. For edgeR, the assay of *zinbwave* output is converted to *DGEList* object using *DGEList* function. The functions *glmFit* and *glmWeightedF* are then used in order. For *DESeq2*, *DESeqDataSet* is used to create an object from the output of the function *zinbwave*. Then, the functions *DESeq* and *lfcShrink* are used in order on the output to obtain the DE analysis results. The options, *sfType="poscounts"*, *useT=TRUE*, *minmu=1e-6* were used for *DESeq2* function as described in *zinbwave* vignettes page. We found that the

performance of DESeq2 tended to improve when the pseudocounts of one were added to the '*counts*' field of the *zinbwave* output before creating the object using the function *DESeqDataSet*.

- *Pseudobulk*[18] DE approach: Raw read counts for each gene are aggregated across the cells of a given group within each batch/dataset. Each collapsed sample will be treated like a single bulk sample for that group. Any DE method designed for bulk RNA-seq data can be applied to the pseudobulk data for identifying DE genes.

- *LogN*: DE workflows including "LogN" used the "LogNormalize" function in Seurat to log-scale the raw count data.

- *Meta-analysis (FEM/REM)*[19]: *FEM* and *REM* combine effect size of each gene from multiple batches. Both approaches are implemented using *get.FEM2* and *get.REM2* functions of *MetaDE* R package[20] (ver.1.0.5) as. *get.ES* function calculates the variances and effect sizes for each gene and batch for those two functions. We have manually set functions not to run additional filtering and imputation steps. This function was included in our github page. We have run *ind.cal.ES.core* function on log-normalized count data. When *DESeq2* was used for each batch, "log2FoldChange" output in each batch was used as effect size, and "lfcSE" was converted to variance for FEM/REM meta-analysis. The output of *FEM/REM* include the meta-analysis *p*-values and average effect sizes for all genes.

- *Meta-analysis (Fisher/wFisher)*[21,22]: We used the function *wFisher* from metapro R package. For each gene, DE *p*-values and its signs from each batch are used as input for "p" and "eff.sign" parameters, respectively. Additional input is the vector of expressed cell counts in each batch, which is passed to "weight" parameter. The output consists of meta-analysis *p*-value and DE direction (+/-) for each gene. We converted "+" and "-" DE direction to +1 and -1, respectively and used them as the sign of each gene. We used *wFisher* for three DE methods: DESeq2, edgeR and limmatrend. While *DESeq2* and *edgeR* are applied to raw count data, *limmatrend* is applied to log-normalized count data. For prognostic gene selection by integrating the five survival analysis results, we used the number of samples from each microarray dataset as "weight" and sign of log Hazard ratio as "eff.sign".

- *scVI*[23] (via Python library *scvi-tools* version 0.17.3): Single-cell variational inference is a part of packaged Python library *scvi-tools* which provide the probabilistic representation and analysis of scRNA-seq data. This method uses deep neural networks with stochastic optimization to aggregate information across similar cells and genes to approximate the distributions that underlie the observed expression values, while accounting for batch effects and limited sensitivity[23]. For this methods, we used the Python pipeline *Scanpy* (version 1.9.1) to preprocess the data. The function *pp.normalize_total* is used to normalize the library size for each cell (sum 10,000) and the function *pp.log1p* is used to perform log-transformation. The top 2000 highly variable genes are extracted for reference during the training process. We use the function *model.SCVI* to build a *scVI* model with a network structure including: input layer, three hidden 1024-node layers for encoding, one latent space with 128 nodes, another three

hidden 1024-node layers for decoding and final output layer (default: single hidden layers). The corrected data is obtained via the function *get_normalized_expression* of the trained model.

- *scGen*[24] (Python library version 2.1.0): This transfer learning method combines variational auto-encoders (VAEs) and latent space vector arithmetic to model and predict missing values of single-cell expression data[24]. A deep neural network model is trained on the observed data using VAEs, and then is used to predict the distribution of the query dataset. The pipeline *Scanpy* functions *pp.normalize_total* and *pp.log1p* are used to normalize and log-scale the count data before training a *scGen* network in 3,000 epochs with the batch size of 32 samples (cells) without an early stopping option to exhaustively explore the latent space. Finally, we obtain the BEC expression matrix from the function *batch_removal* of the trained model.

- S*canorama*[25] (Python library version 1.7.2): The basic concept of this approach is to consider each batch as a perspective of the *future corrected* data. Then, the batches are integrated in the order of restoring the *panorama* view of the corrected data. An approximate nearest neighbor search is used to identify mutually linked cells across batches with the expression in the reduced dimension using approximate singular value decomposition (SVD) in a pair-by-pair manner. The order of matching is determined by the ratio of matching cells between batches. The pipeline *Scanpy* functions *pp.normalize_total* and *pp.log1p* are used to normalize and log-scale the count data. Scanorama works seamlessly with the *Scanpy* pipeline since the normalized and log-transformed data obtained using *Scanpy* is fed directly to the function *scanorama.correct_scanpy*. Then, we have the output of the corrected data. Note that the order of genes and cells may be shuffled after the correction.

- *RISC*[26] (R package ver. 1.5.0): *RISC* integrates scRNA-seq datasets using principal components (PCs) via principal component regression (PCR) model. It takes advantage of the natural compatibility of eigenvectors between PCR model and dimension reduction for accurate integration of scRNA-seq datasets[26]. Before running *RISC* data integration, each batch/dataset should be preprocessed using *scFilter*, *scNormalize* and *scDisperse* function from the *RISC* package. We used *is.Filter=F* parameter for scFilter function to obtain the integrated data of the same size with the original raw count data. Then, we have merged preprocessed data into a list and run *InPlot* function to check how the PCs explain the variance for data integration. We have used *nPC=40* and *minPC=16*. The Std.cut parameter is recommended to be 0.85 ~ 0.9 for small-scale data (total cells < 10,000) and 0.9 ~ 0.98 for large-scale data (total cells > 10,000). We have used $Std.cut = 0.85 + 0.025 * log_{10}(total\ cells)/100)$ for small-scale data (total cells < 10,000) and $Std.cut = 0.9 + 0.04 * log_{10}(total\ cells)/10000)$ for large-scale data (total cells > 10,000). The *InPlot* function provides three different scores for each batch, “Cluster Num”, “Stv by PCs” and “Kolmogorov-Smirnov”. The batch with the largest score, weighted in the order of Cluster Num, Stv by PCs and Kolmogorov-Smirnov was manually selected as the reference batch for integration. We have rearranged the reference dataset to the first element of the merged list and passed it as input for the *scMultiIntegrate* function. The corrected data are stored in *logcount* slot of *scMultiIntegrate* output.

Supplementary Figures

A motivation for using pAUPR and $F_{0.5}$-scores

In the precision-recall results for the MCA T-cell data (Supplementary Fig. 3h), edgeR showed a lower precision compared to LogN_FEM and MAST in the area of recall < 0.5; however, the precision of edgeR was much higher for recall > 0.5 (Supplementary Fig. 1a). As a result, the AUPR of edgeR which used the whole recall area was higher than those of LogN and MAST. This pattern was even clearer when we analyzed MCA B-cells (Supplementary Fig. 1b, c). We assumed LogN_FEM and MAST in these results performed better than edgeR because it is of particular important to identify a small number of DE genes (or markers) in the analysis of noisy and sparse scRNA-seq data. For a similar reason, we used $F_{0.5}$-scores.

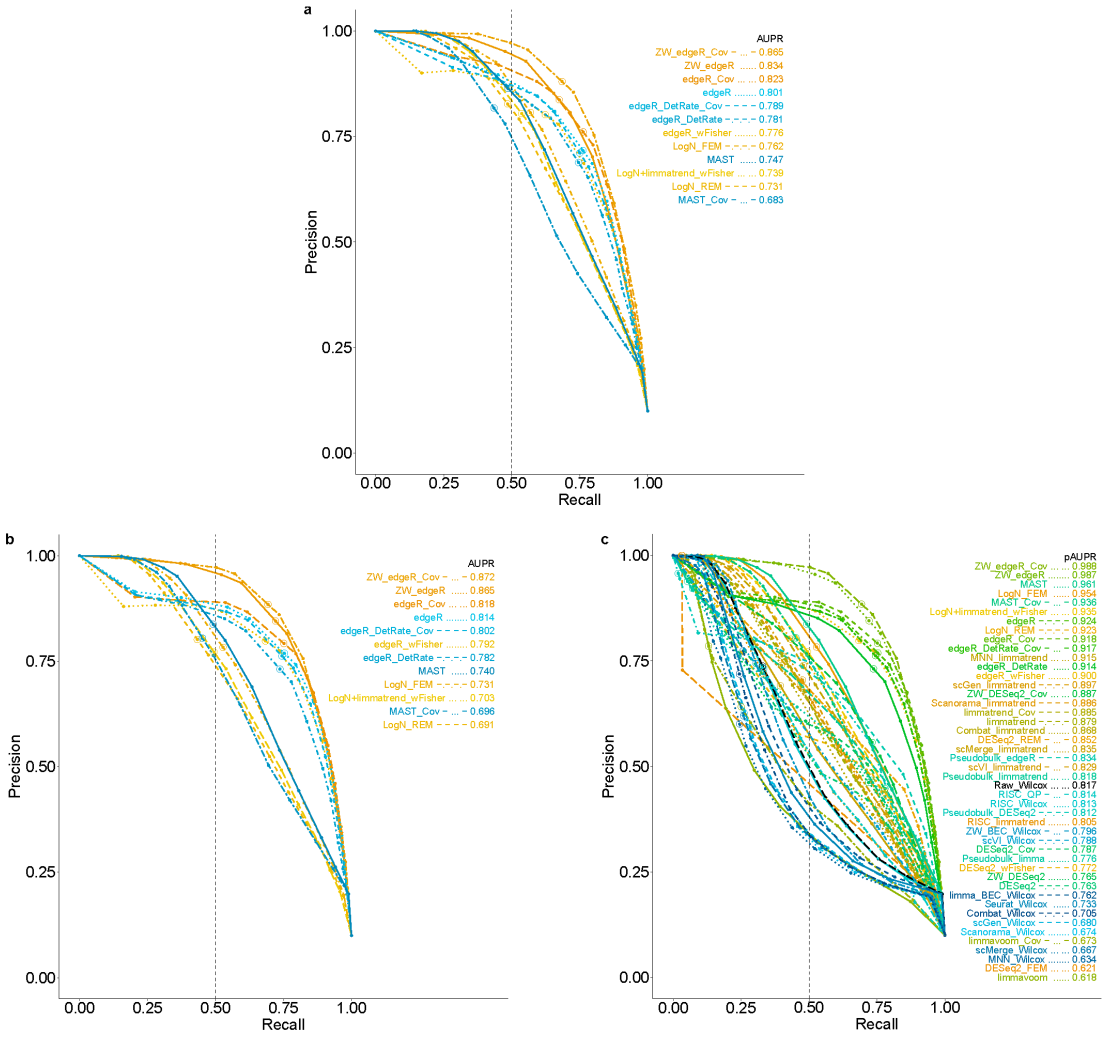

Figure S1. Model-free simulation results for MCA T-cells and B-cells. **a** Areas under the precision-recall (AUPRs) of selected differential expression (DE) workflows for T-cells. AUPRs of selected workflows for **b** B-cells and **c** partial AUPR (pAUPR) results for all DE workflows. The precision-recall pairs that correspond to q-value = 0.05 in each DE workflow are circled.

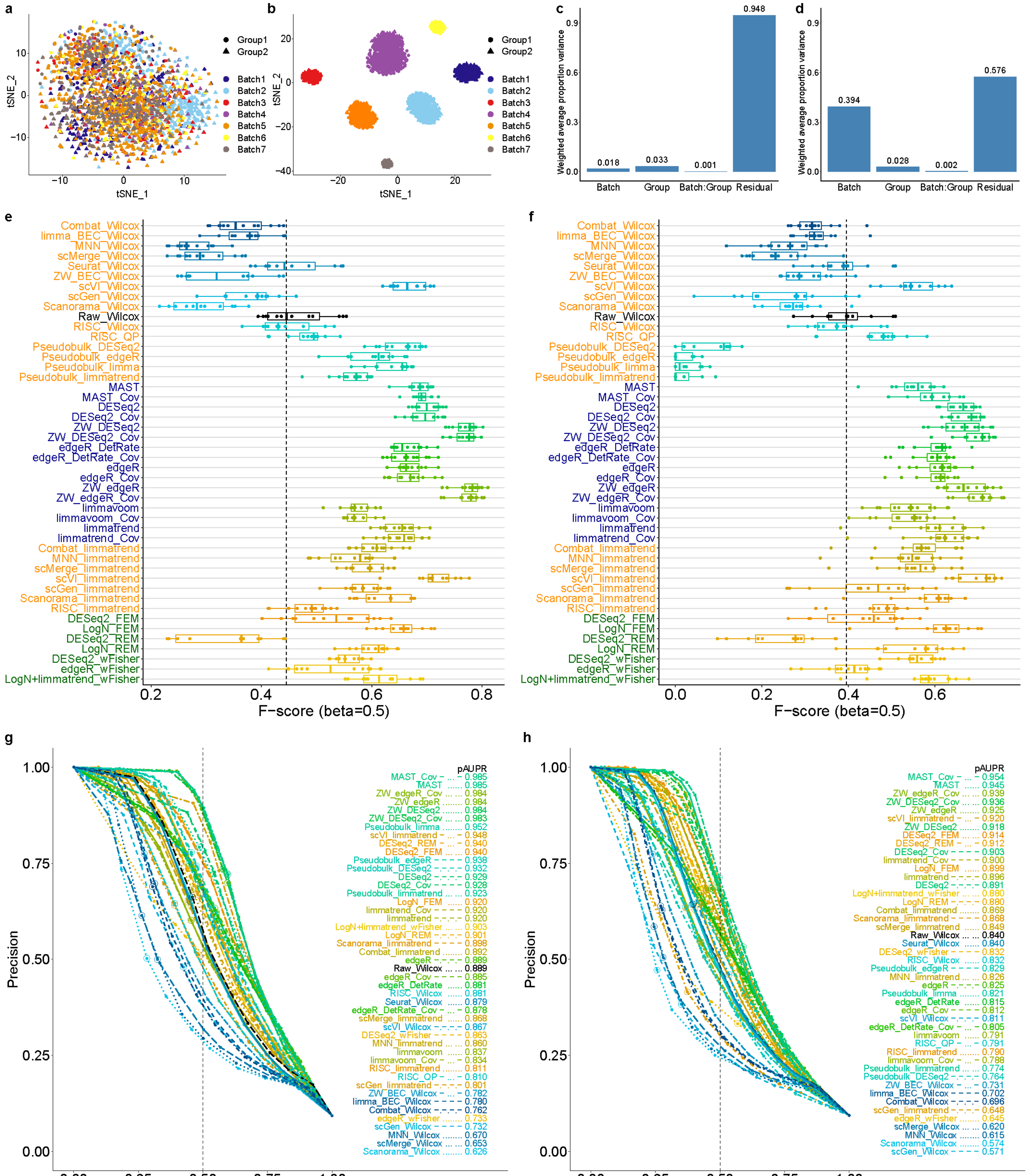

a
b
c
d
e
f
g
h
tSNE_1
tSNE_2
Group1
Group2
Batch1
Batch2
Batch3
Batch4
Batch5
Batch6
Batch7
Weighted average proportion variance
Batch
Group
Batch:Group
Residual
0.018
0.033
0.001
0.948
0.394
0.028
0.002
0.576
Combat_Wilcox
limma_BEC_Wilcox
MNN_Wilcox
scMerge_Wilcox
Seurat_Wilcox
ZW_BEC_Wilcox
scVI_Wilcox
scGen_Wilcox
Scanorama_Wilcox
Raw_Wilcox
RISC_Wilcox
RISC_QP
Pseudobulk_DESeq2
Pseudobulk_edgeR
Pseudobulk_limma
Pseudobulk_limmatrend
MAST
MAST_Cov
DESeq2
DESeq2_Cov
ZW_DESeq2
ZW_DESeq2_Cov
edgeR_DetRate
edgeR_DetRate_Cov
edgeR
edgeR_Cov
ZW_edgeR
ZW_edgeR_Cov
limmavoom
limmavoom_Cov
limmatrend
limmatrend_Cov
Combat_limmatrend
MNN_limmatrend
scMerge_limmatrend
scVI_limmatrend
scGen_limmatrend
Scanorama_limmatrend
RISC_limmatrend
DESeq2_FEM
LogN_FEM
DESeq2_REM
LogN_REM
DESeq2_wFisher
edgeR_wFisher
LogN+limmatrend_wFisher
F−score (beta=0.5)
Precision
Recall
pAUPR
MAST_Cov 0.985
MAST 0.985
ZW_edgeR_Cov 0.984
ZW_edgeR 0.984
ZW_DESeq2 0.984
ZW_DESeq2_Cov 0.983
Pseudobulk_limma 0.952
scVI_limmatrend 0.948
DESeq2_REM 0.940
DESeq2_FEM 0.940
Pseudobulk_edgeR 0.938
Pseudobulk_DESeq2 0.932
DESeq2 0.929
DESeq2_Cov 0.928
Pseudobulk_limmatrend 0.923
LogN_FEM 0.920
limmatrend_Cov 0.920
limmatrend 0.920
LogN+limmatrend_wFisher 0.903
LogN_REM 0.901
Scanorama_limmatrend 0.898
Combat_limmatrend 0.892
edgeR 0.889
Raw_Wilcox 0.889
edgeR_Cov 0.885
edgeR_DetRate 0.881
RISC_Wilcox 0.881
Seurat_Wilcox 0.879
edgeR_DetRate_Cov 0.878
scMerge_limmatrend 0.868
scVI_Wilcox 0.867
DESeq2_wFisher 0.863
MNN_limmatrend 0.860
limmavoom 0.837
limmavoom_Cov 0.834
RISC_limmatrend 0.811
RISC_QP 0.810
scGen_limmatrend 0.801
ZW_BEC_Wilcox 0.782
limma_BEC_Wilcox 0.780
Combat_Wilcox 0.762
edgeR_wFisher 0.733
scGen_Wilcox 0.732
MNN_Wilcox 0.670
scMerge_Wilcox 0.653
Scanorama_Wilcox 0.626
MAST_Cov 0.954
MAST 0.945
ZW_edgeR_Cov 0.939
ZW_DESeq2_Cov 0.936
ZW_edgeR 0.925
scVI_limmatrend 0.920
ZW_DESeq2 0.918
DESeq2_FEM 0.914
DESeq2_REM 0.912
DESeq2_Cov 0.903
limmatrend_Cov 0.900
LogN_FEM 0.899
limmatrend 0.896
DESeq2 0.891
LogN+limmatrend_wFisher 0.880
LogN_REM 0.880
Combat_limmatrend 0.869
Scanorama_limmatrend 0.868
scMerge_limmatrend 0.849
Raw_Wilcox 0.840
Seurat_Wilcox 0.840
DESeq2_wFisher 0.832
RISC_Wilcox 0.832
Pseudobulk_edgeR 0.829
MNN_limmatrend 0.826
edgeR 0.825
Pseudobulk_limma 0.821
edgeR_DetRate 0.815
edgeR_Cov 0.812
scVI_Wilcox 0.811
edgeR_DetRate_Cov 0.805
limmavoom 0.791
RISC_QP 0.791
RISC_limmatrend 0.790
limmavoom_Cov 0.788
Pseudobulk_limmatrend 0.774
Pseudobulk_DESeq2 0.764
ZW_BEC_Wilcox 0.731
limma_BEC_Wilcox 0.702
Combat_Wilcox 0.696
scGen_limmatrend 0.648
edgeR_wFisher 0.645
scMerge_Wilcox 0.620
MNN_Wilcox 0.615
Scanorama_Wilcox 0.574
scGen_Wilcox 0.571

Figure S2. Model-based simulation results for moderate depths, seven batches and high sparsity (zero rate > 80%). Scatter plots (tSNE) of seven batches for **a** small and **b** large batch effects. Principal component variance analysis results representing **c** small and **d** large batch effects. $F_{0.5}$-scores for 46 differential expression workflows for **e** small and **f** large batch effects. Results for six cell proportion scenarios (12 instances in total: six for upregulated genes and six for downregulated genes) are represented as boxplots; the lower, center and upper bars represent the 25th , 50th and 75th percentiles, respectively, and the whiskers represent ± 1.5 × interquartile range. The vertical dotted lines (black) indicate the median $F_{0.5}$-score of Wilcoxon test (Raw_Wilcox). Precision-recall curves for **g** small and **f** large batch effects. The partial areas under the curve for recall rate < 0.5 (pAUPRs) are computed and sorted in descending order in the legends. The vertical dotted lines (black) indicate the recall rate of 0.5. The precision-recall pairs that correspond to *q*-value = 0.05 in each differential expression (DE) workflow are circled. Here, we used slightly reduced effect sizes of DE genes compared to the two-batch case to appropriately compare the relative performance of benchmark methods. n=2400 cells were used for each test case.

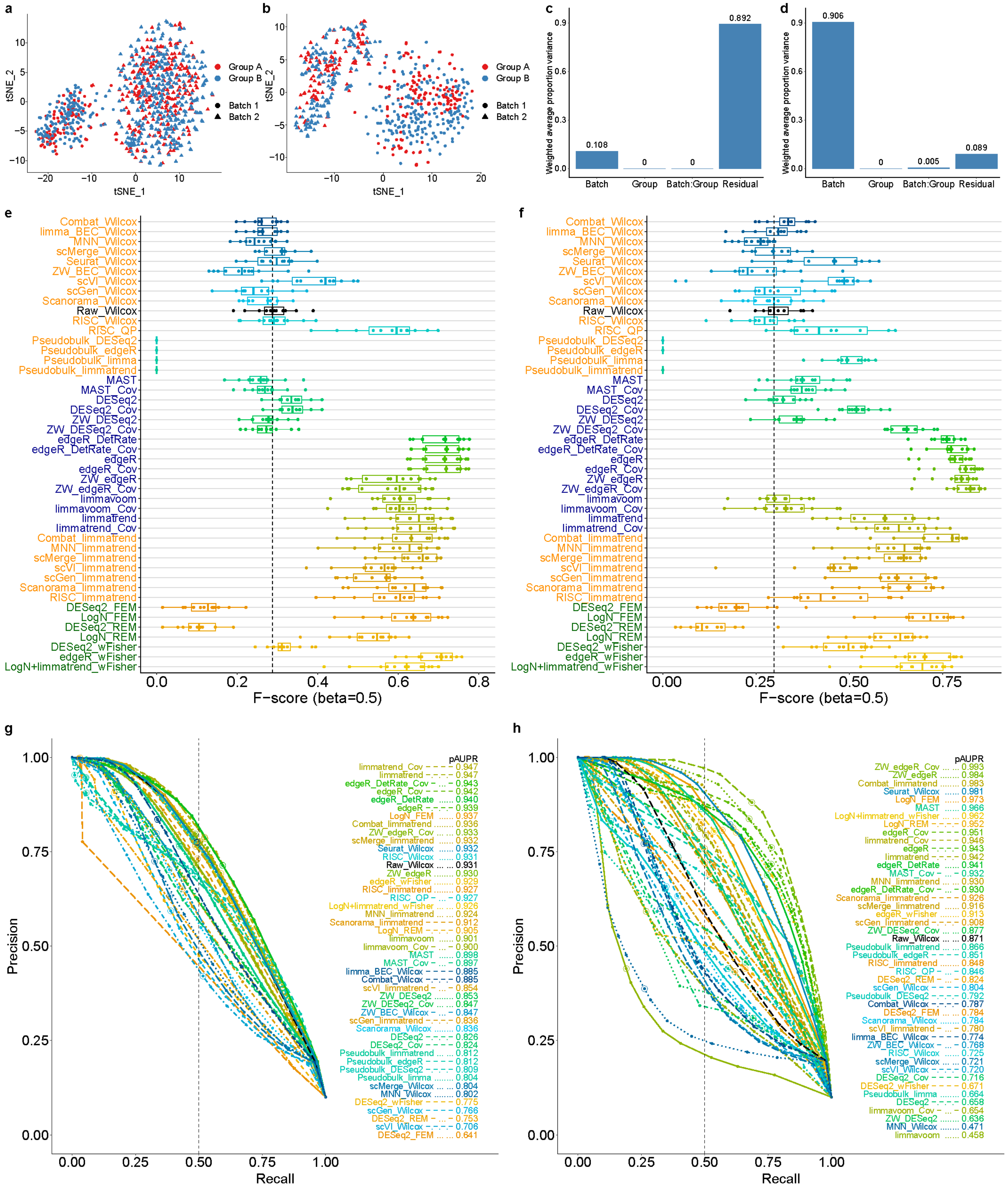
a
b
c
d
e
f
g
h
tSNE_1
tSNE_2
Group A
Group B
Batch 1
Batch 2
Weighted average proportion variance
Batch
Group
Batch:Group
Residual
0.108
0
0
0.892
0.906
0
0.005
0.089
F−score (beta=0.5)
Combat_Wilcox
limma_BEC_Wilcox
MNN_Wilcox
scMerge_Wilcox
Seurat_Wilcox
ZW_BEC_Wilcox
scVI_Wilcox
scGen_Wilcox
Scanorama_Wilcox
Raw_Wilcox
RISC_Wilcox
RISC_QP
Pseudobulk_DESeq2
Pseudobulk_edgeR
Pseudobulk_limma
Pseudobulk_limmatrend
MAST
MAST_Cov
DESeq2
DESeq2_Cov
ZW_DESeq2
ZW_DESeq2_Cov
edgeR_DetRate
edgeR_DetRate_Cov
edgeR
edgeR_Cov
ZW_edgeR
ZW_edgeR_Cov
limmavoom
limmavoom_Cov
limmatrend
limmatrend_Cov
Combat_limmatrend
MNN_limmatrend
scMerge_limmatrend
scVI_limmatrend
scGen_limmatrend
Scanorama_limmatrend
RISC_limmatrend
DESeq2_FEM
LogN_FEM
DESeq2_REM
LogN_REM
DESeq2_wFisher
edgeR_wFisher
LogN+limmatrend_wFisher
Precision
Recall
pAUPR
limmatrend_Cov 0.947
limmatrend 0.947
edgeR_DetRate_Cov 0.943
edgeR_Cov 0.942
edgeR_DetRate 0.940
edgeR 0.939
LogN_FEM 0.937
Combat_limmatrend 0.936
ZW_edgeR_Cov 0.933
scMerge_limmatrend 0.932
Seurat_Wilcox 0.932
RISC_Wilcox 0.931
Raw_Wilcox 0.931
ZW_edgeR 0.930
edgeR_wFisher 0.929
RISC_limmatrend 0.927
RISC_QP 0.927
LogN+limmatrend_wFisher 0.926
MNN_limmatrend 0.924
Scanorama_limmatrend 0.912
LogN_REM 0.905
limmavoom 0.901
limmavoom_Cov 0.900
MAST 0.898
MAST_Cov 0.897
limma_BEC_Wilcox 0.885
Combat_Wilcox 0.885
scVI_limmatrend 0.854
ZW_DESeq2 0.853
ZW_DESeq2_Cov 0.847
ZW_BEC_Wilcox 0.847
scGen_limmatrend 0.836
Scanorama_Wilcox 0.836
DESeq2 0.826
DESeq2_Cov 0.824
Pseudobulk_limmatrend 0.812
Pseudobulk_edgeR 0.812
Pseudobulk_DESeq2 0.809
Pseudobulk_limma 0.804
scMerge_Wilcox 0.804
MNN_Wilcox 0.802
DESeq2_wFisher 0.775
scGen_Wilcox 0.766
DESeq2_REM 0.753
scVI_Wilcox 0.706
DESeq2_FEM 0.641
pAUPR
ZW_edgeR_Cov 0.993
ZW_edgeR 0.984
Combat_limmatrend 0.983
Seurat_Wilcox 0.981
LogN_FEM 0.973
MAST 0.966
LogN+limmatrend_wFisher 0.962
LogN_REM 0.952
edgeR_Cov 0.951
limmatrend_Cov 0.946
edgeR 0.943
limmatrend 0.942
edgeR_DetRate 0.941
MAST_Cov 0.932
MNN_limmatrend 0.930
edgeR_DetRate_Cov 0.930
Scanorama_limmatrend 0.926
scMerge_limmatrend 0.916
edgeR_wFisher 0.913
scGen_limmatrend 0.908
ZW_DESeq2_Cov 0.877
Raw_Wilcox 0.871
Pseudobulk_limmatrend 0.866
Pseudobulk_edgeR 0.851
RISC_limmatrend 0.848
RISC_QP 0.846
DESeq2_REM 0.824
scGen_Wilcox 0.804
Pseudobulk_DESeq2 0.792
Combat_Wilcox 0.787
DESeq2_FEM 0.784
Scanorama_Wilcox 0.784
scVI_limmatrend 0.780
limma_BEC_Wilcox 0.774
ZW_BEC_Wilcox 0.768
RISC_Wilcox 0.725
scMerge_Wilcox 0.721
scVI_Wilcox 0.720
DESeq2_Cov 0.716
DESeq2_wFisher 0.671
Pseudobulk_limma 0.664
DESeq2 0.658
limmavoom_Cov 0.654
ZW_DESeq2 0.636
MNN_Wilcox 0.471
limmavoom 0.458

Figure S3. Model-free simulation results. Scatter plots for **a** pancreatic alpha-cell and **b** MCA T-cell data. Principal component variance analysis results for **c** pancreatic alpha-cell and **d** MCA T-cell data, representing small and large batch effects, respectively. The $F_{0.5}$-scores for **e** pancreatic alpha-cell and **f** MCA T-cell data. Results for six cell proportion scenarios (12 instances in total: six for upregulated genes and six for downregulated genes) are represented as boxplots; the lower, center and upper bars represent the 25th , 50th and 75th percentiles, respectively, and the whiskers represent $\pm 1.5 \times$ interquartile range. The vertical dotted lines (black) indicate the median $F_{0.5}$-score of Wilcoxon test (Raw_Wilcox). Precision-recall curves for the 46 differential expression (DE) workflows for **g** pancreatic alpha-cell and **h** MCA T-cell data. The partial areas under the precision-recall curve for recall rate < 0.5 (pAUPR) are computed and sorted in descending order in the legends. The vertical dotted lines (black) indicate the recall rate of 0.5. The precision-recall pairs that correspond to *q*-value = 0.05 in each DE workflow are circled. n=900 and 3059 cells were used for (**e**, **g**) pancreatic alpha-cell and (**f**, **h**) MCA T-cell data, respectively.

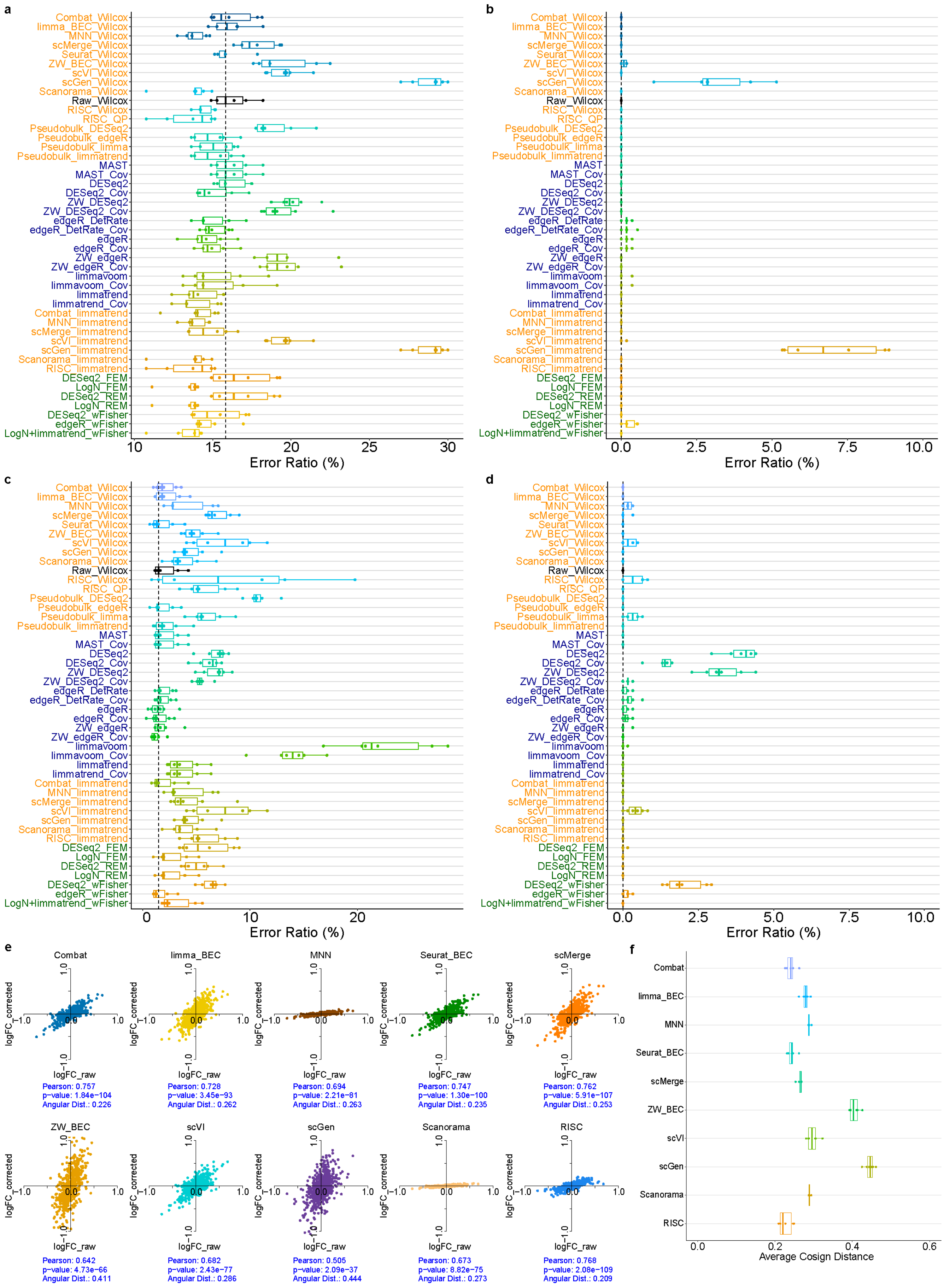

a
Combat_Wilcox
limma_BEC_Wilcox
MNN_Wilcox
scMerge_Wilcox
Seurat_Wilcox
ZW_BEC_Wilcox
scVI_Wilcox
scGen_Wilcox
Scanorama_Wilcox
Raw_Wilcox
RISC_Wilcox
RISC_QP
Pseudobulk_DESeq2
Pseudobulk_edgeR
Pseudobulk_limma
Pseudobulk_limmatrend
MAST
MAST_Cov
DESeq2
DESeq2_Cov
ZW_DESeq2
ZW_DESeq2_Cov
edgeR_DetRate
edgeR_DetRate_Cov
edgeR
edgeR_Cov
ZW_edgeR
ZW_edgeR_Cov
limmavoom
limmavoom_Cov
limmatrend
limmatrend_Cov
Combat_limmatrend
MNN_limmatrend
scMerge_limmatrend
scVI_limmatrend
scGen_limmatrend
Scanorama_limmatrend
RISC_limmatrend
DESeq2_FEM
LogN_FEM
DESeq2_REM
LogN_REM
DESeq2_wFisher
edgeR_wFisher
LogN+limmatrend_wFisher
10
15
20
25
30
Error Ratio (%)
b
0.0
2.5
5.0
7.5
10.0
Error Ratio (%)
c
0
10
20
Error Ratio (%)
d
0.0
2.5
5.0
7.5
10.0
Error Ratio (%)
e
Combat
logFC_corrected
logFC_raw
Pearson: 0.757
p-value: 1.84e−104
Angular Dist.: 0.226
limma_BEC
Pearson: 0.728
p-value: 3.45e−93
Angular Dist.: 0.262
MNN
Pearson: 0.694
p-value: 2.21e−81
Angular Dist.: 0.263
Seurat_BEC
Pearson: 0.747
p-value: 1.30e−100
Angular Dist.: 0.235
scMerge
Pearson: 0.762
p-value: 5.91e−107
Angular Dist.: 0.253
ZW_BEC
Pearson: 0.642
p-value: 4.73e−66
Angular Dist.: 0.411
scVI
Pearson: 0.682
p-value: 2.43e−77
Angular Dist.: 0.286
scGen
Pearson: 0.505
p-value: 2.09e−37
Angular Dist.: 0.444
Scanorama
Pearson: 0.673
p-value: 8.82e−75
Angular Dist.: 0.273
RISC
Pearson: 0.768
p-value: 2.08e−109
Angular Dist.: 0.209
f
Combat
limma_BEC
MNN
Seurat_BEC
scMerge
ZW_BEC
scVI
scGen
Scanorama
RISC
0.0
0.2
0.4
0.6
Average Cosign Distance

Figure S4. Distortion analysis for differential expression (DE) workflows. a Proportion of DE genes that altered their signs by each DE workflow (error ratios) for model-based simulation with a low depth (two batches; large batch effects; depth-4). **b** Error ratios for the model-based simulation for only significantly detected DE genes (q-value < 0.05). The vertical dotted lines (black) indicate the median error ratio of Wilcoxon test (Raw_Wilcox). **c** Error ratios for MCA T-cell (model-free) simulation data. **d** Error ratios for the MCA data for only significantly detected DE genes. **e** A scatterplot of the logFC values for the model-based simulation data with a low depth (depth-4) before (logFC_raw) and after (logFC_corrected) applying batch-effect correction (BEC) methods: Combat, limma (limma_BEC), MNNCorrect, Seurat_BEC, scMerge, ZINB-WaVE (ZW_BEC), scVI, scGen, Scanorama and RISC. Pearson correlation, its p-value and the angular cosine distance (Angular Dist) of scatter plot are shown for each BEC method. **f** The distortion levels for the low depth data as measured by the angular cosine distance from the logFC scatterplot for six cell proportion scenarios. The lower, center and upper bars of each boxplot represent the 25th , 50th and 75th percentiles, respectively, and the whiskers represent $\pm 1.5 \times$ interquartile range. n=1000 cells were used in **a**, **b**, **e**, **f**, and n=624 cells were used in **c** and **d**.

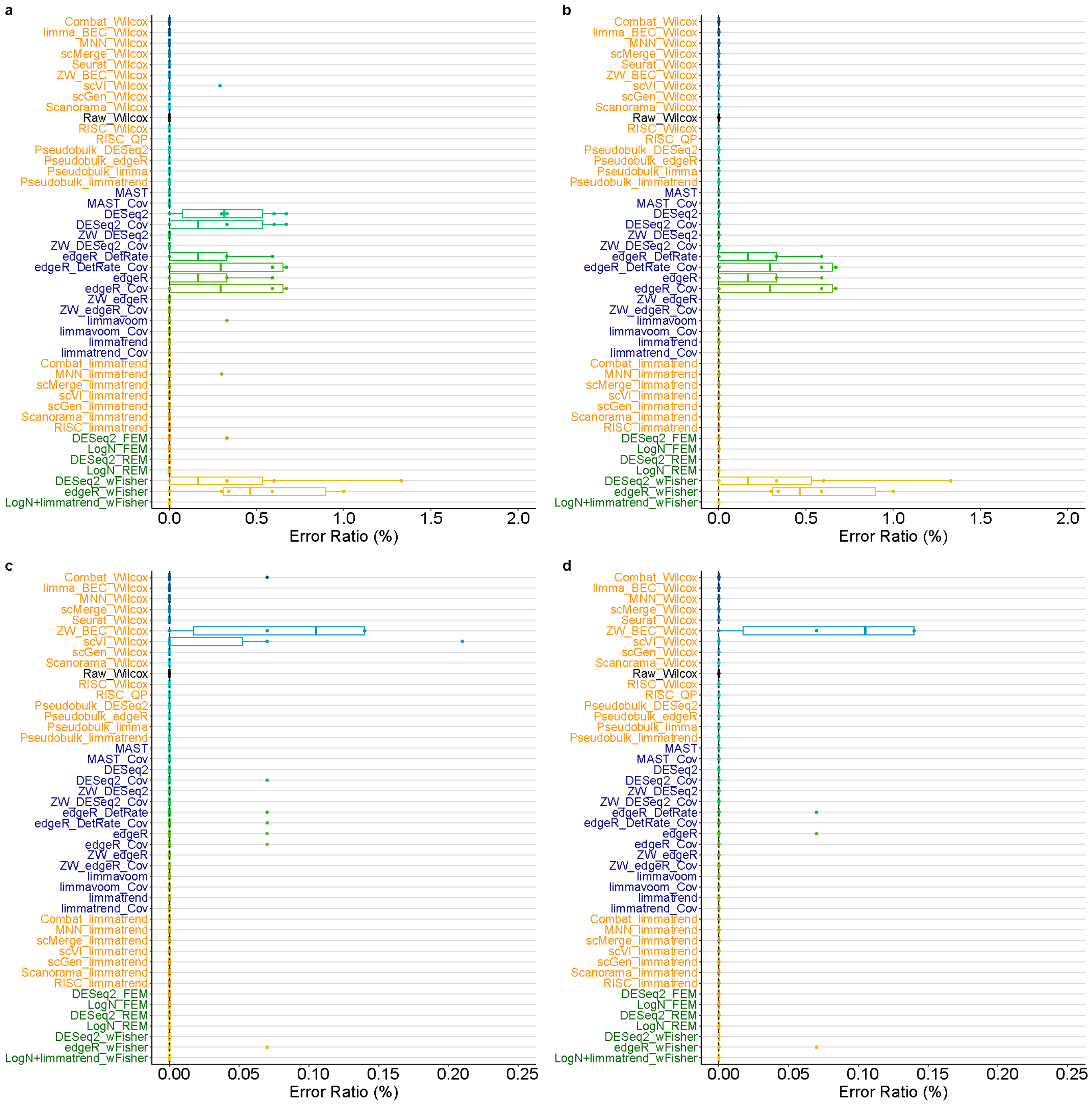

Figure S5. Error ratios for differentially expressed genes with or without FC threshold ($|logFC| > 0.5$). Error ratios for model-based simulation **a** for q-value < 0.05 only and **b** for both thresholds q-value < 0.05 and $|logFC| > 0.5$. Error ratios for pancreatic alpha-cell simulation **c** for q-value < 0.05 only and **d** for both thresholds q-value < 0.05 and $|logFC| > 0.5$. The lower, center and upper bars of each boxplot represent the 25th , 50th and 75th percentiles, respectively, and the whiskers represent ± 1.5 × interquartile range. n=1050 cells were used in **a**, **b** and n=900 cells were used in **c**, **d**.

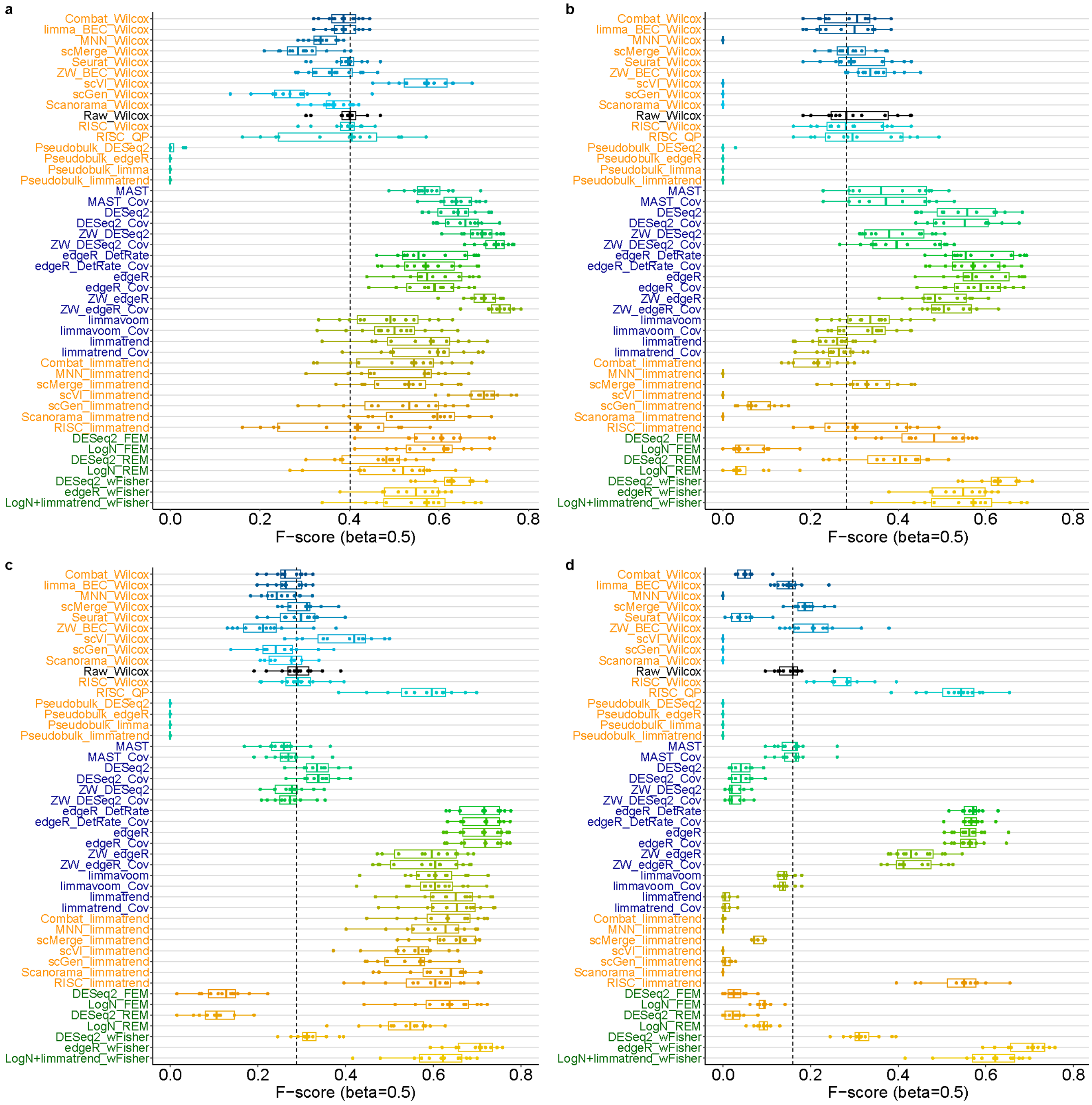

Figure S6. $F_{0.5}$-scores with or without FC threshold ($|logFC| > 0.5$). $F_{0.5}$-scores for model-based simulation **a** for q-value < 0.05 only and **b** for both thresholds q-value < 0.05 and $|logFC| > 0.5$. $F_{0.5}$-scores for pancreatic alpha-cell simulation **c** for q-value < 0.05 only and **d** for both thresholds q-value < 0.05 and $|logFC| > 0.5$. Results for six cell proportion scenarios (12 instances in total: six for upregulated genes and six for downregulated genes) are represented as boxplots; the lower, center and upper bars represent the 25th , 50th and 75th percentiles, respectively, and the whiskers represent ± 1.5 × interquartile range. The vertical dotted lines (black) indicate the median $F_{0.5}$-score of Wilcoxon test (Raw_Wilcox). n=1050 cells were used in **a**, **b** and n=900 cells were used in **c**, **d**.

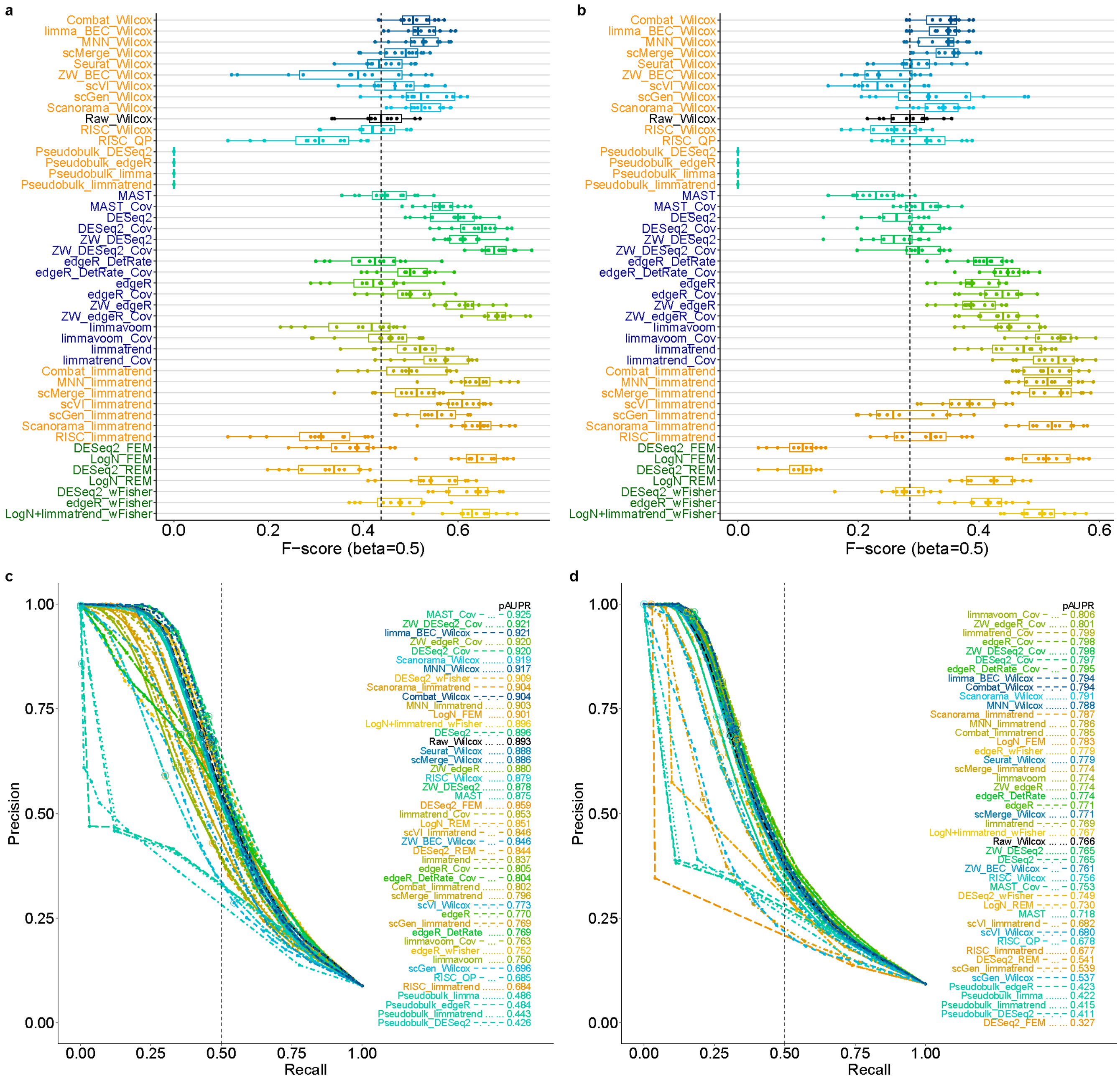

Figure S7. Model-based simulation results for less sparse data (zero rate: 40%) with large batch effects. $F_{0.5}$-scores for 46 differential expression (DE) workflows for **a** moderate and **b** low (depth-4) depths. Results for six cell proportion scenarios (12 instances in total: six for upregulated genes and six for downregulated genes) are represented as boxplots; the lower, center and upper bars represent the 25th, 50th and 75th percentiles, respectively, and the whiskers represent $\pm$ 1.5 $\times$ interquartile range. The vertical dotted lines (black) indicate the median $F_{0.5}$-score of Wilcoxon test (Raw_Wilcox). Precision-recall curves for **c** moderate and **d** low depths. The partial areas under the curve for recall rate < 0.5 (pAUPRs) are computed and sorted in descending order in the legends. The vertical dotted lines (black) indicate the recall rate of 0.5. The precision-recall pairs that correspond to q-value = 0.05 in each DE workflow are circled. n=1050 cells were used in all cases **a-d**.

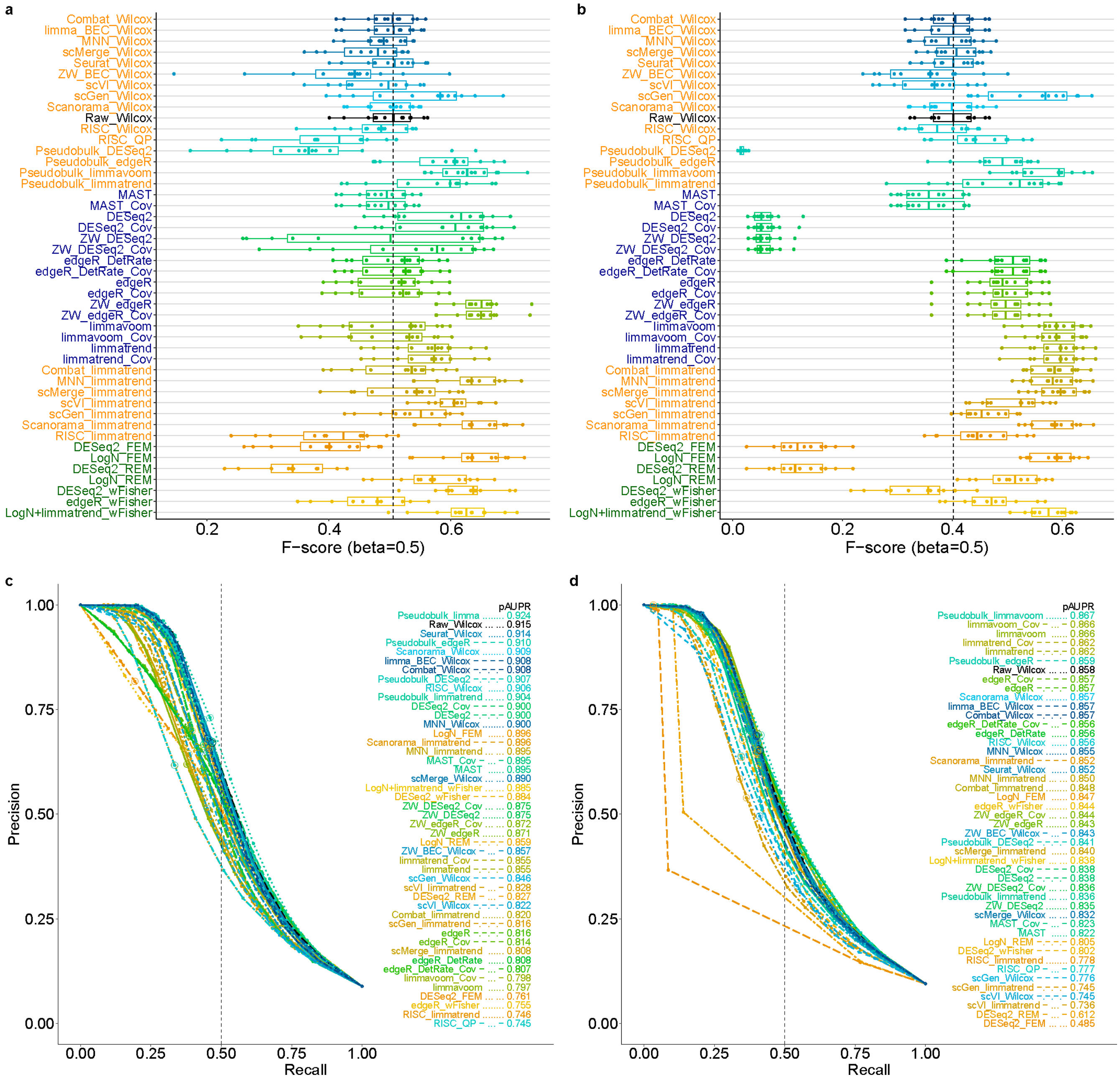

Figure S8. Model-based simulation results for less sparse data (zero rate: 40%) with small batch effects. $F_{0.5}$-scores for 46 differential expression (DE) workflows for **a** moderate and **b** low (depth-4) depths. Results for six cell proportion scenarios (12 instances in total: six for upregulated genes and six for downregulated genes) are represented as boxplots; the lower, center and upper bars represent the 25th , 50th and 75th percentiles, respectively, and the whiskers represent ± 1.5 × interquartile range. The vertical dotted lines (black) indicate the median $F_{0.5}$-score of Wilcoxon test (Raw_Wilcox). Precision-recall curves for **c** moderate and **d** low depths. The partial areas under the curve for recall rate < 0.5 (pAUPRs) are computed and sorted in descending order in the legends. The vertical dotted lines (black) indicate the recall rate of 0.5. The precision-recall pairs that correspond to q-value = 0.05 in each DE workflow are circled. n=1050 cells were used in all cases **a-d**.

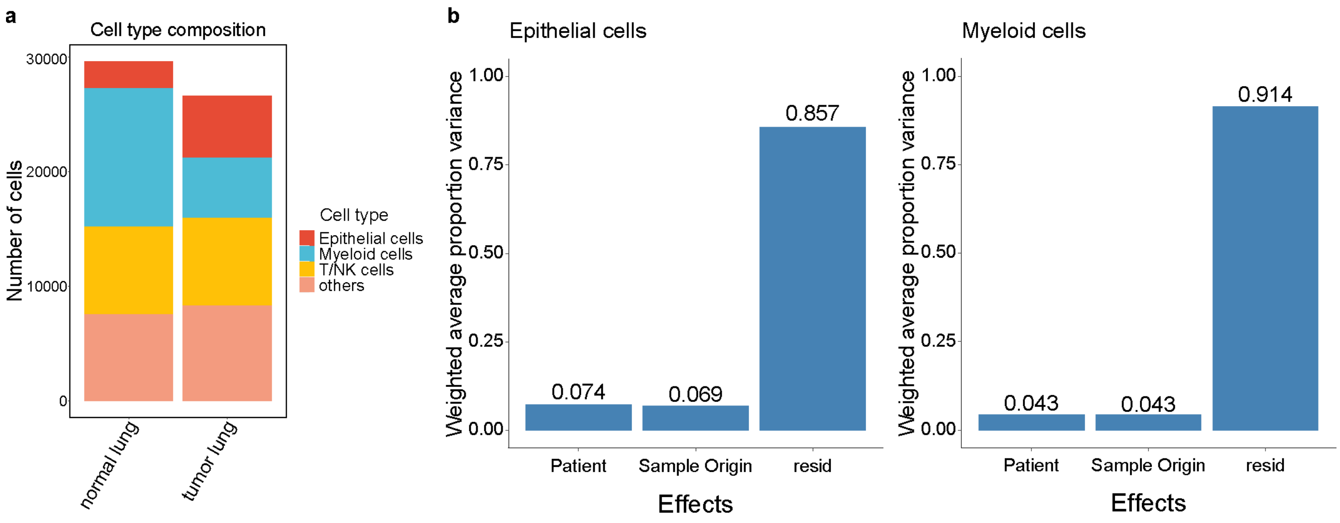

Figure S9. Distribution and batch effects of LUAD scRNA-seq data. a Composition of cell types in normal and tumor lung samples. **b** Principal variance component analysis results for epithelial and myeloid cells. "Patient" represents batch effects factor between patients.

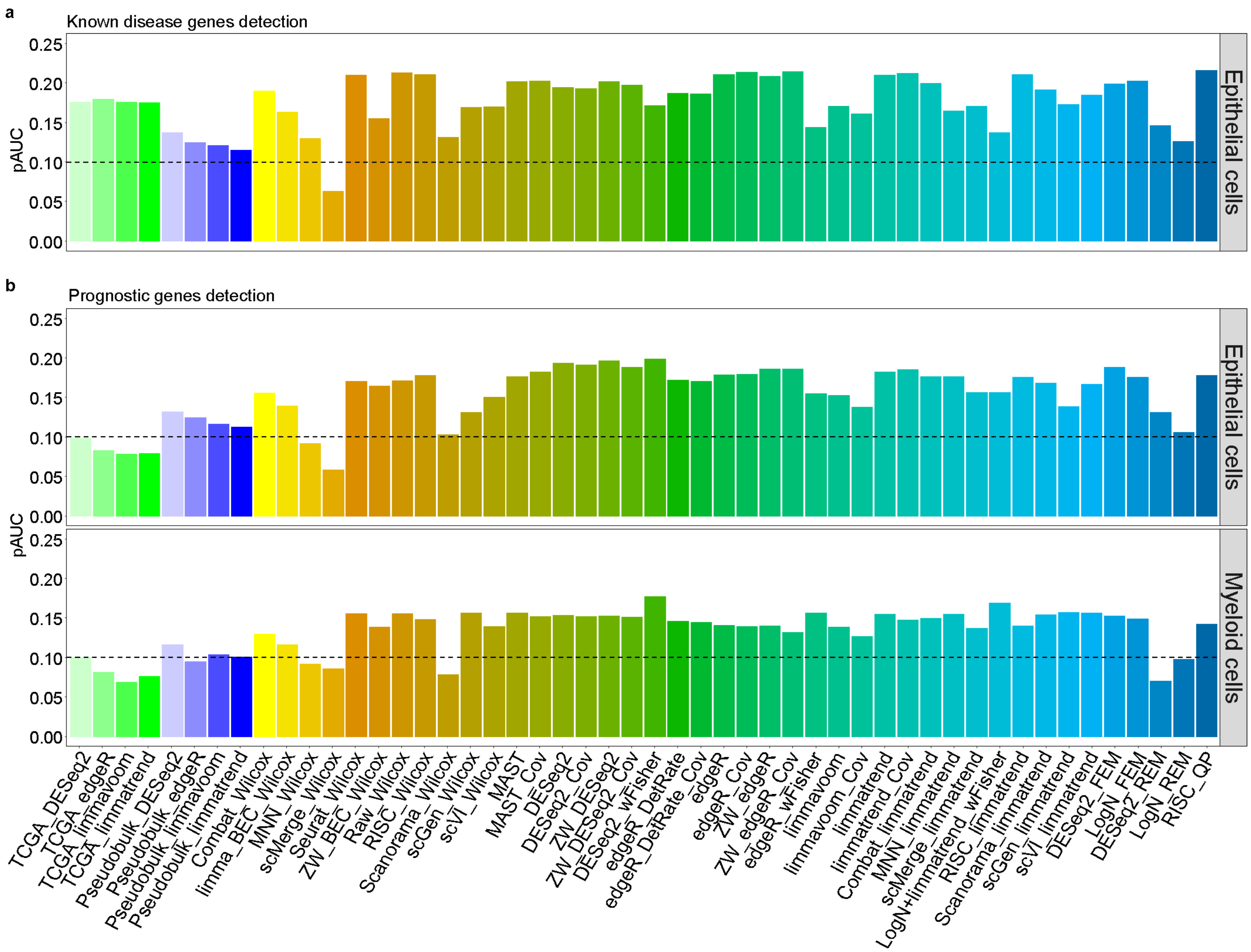

Figure S10. **Partial areas under the curve (pAUC) for standard positive genes detected by differential expression (DE) analysis of scRNA-seq, TCGA lung adenocarcinoma, and pseudobulk data (up to top 20% DE genes). a** pAUC of cumulative disease scores (GDA) from epithelial cell data analysis. **b** pAUC of cumulative counts of prognostic genes from epithelial and myeloid cell data analyses. Black-dashed lines represent pAUCs estimated from the expected numbers of standard positive genes for random gene ranks.

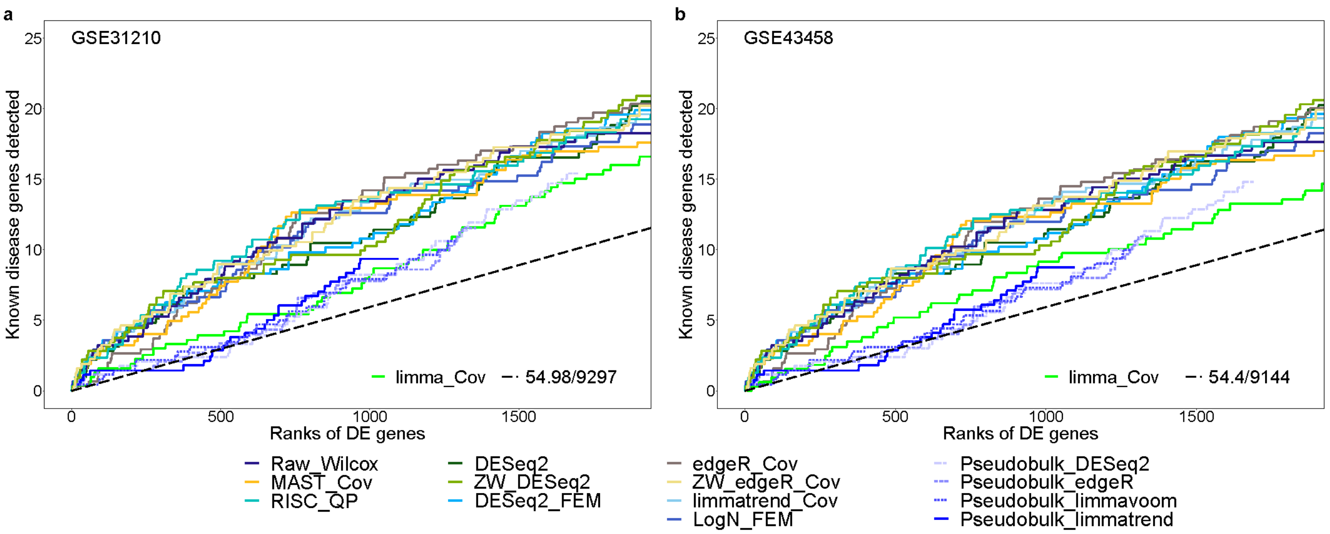

Figure S11. Cumulative scores of known disease genes obtained from analyses of lung adenocarcinoma (LUAD) epithelial cells and two microarray datasets. Cumulative scores of ten scRNA-seq differential expression (DE) methods, four pseudobulk analysis methods, and one bulk sample analysis method (limma) are compared for known disease genes up to top 20% DE gene ranks. Two LUAD microarray expression datasets (**a** GSE31210 and **b** GSE43458) are analyzed (green curves). Black-dashed slopes represent the expected cumulative scores for random gene ranks. 9,144 and 9,297 genes commonly found in both scRNA-seq and microarray datasets (GSE31210 and GSE43458) were analyzed, respectively. n=7728 epithelial cells were used for scRNA-seq data analysis, and n=241 and 110 samples were used from GSE31210 and GSE43458 microarray data analyses, respectively.

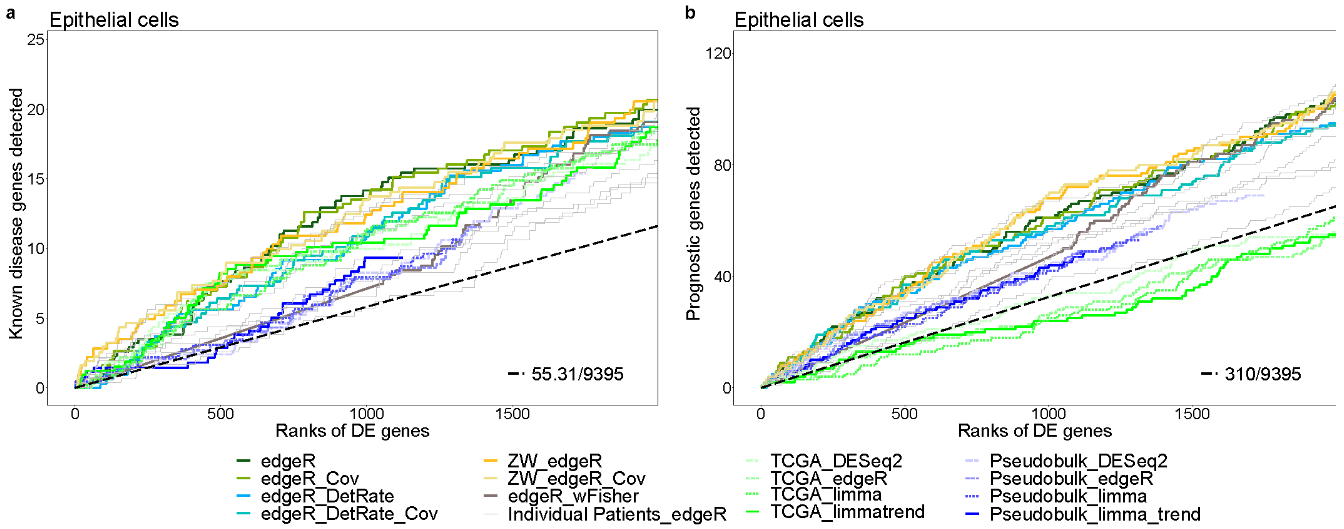

Figure S12. Cumulative scores of known disease genes obtained using scRNA-seq epithelial cells data from individual lung adenocarcinoma (LUAD) patients up to top 20% differential expression (DE) gene ranks. Comparison of cumulative scores for **a** known disease genes and **b** prognostic genes between seven edgeR-based methods, four bulk/pseudobulk analysis methods, and edgeR analyses of the data from seven individual patients. Black-dashed slopes represent the expected cumulative scores for random gene ranks. 9395 genes commonly found in both scRNA-seq epithelial cells (n=7728) and TCGA LUAD RNA-seq datasets (n=585) were analyzed.

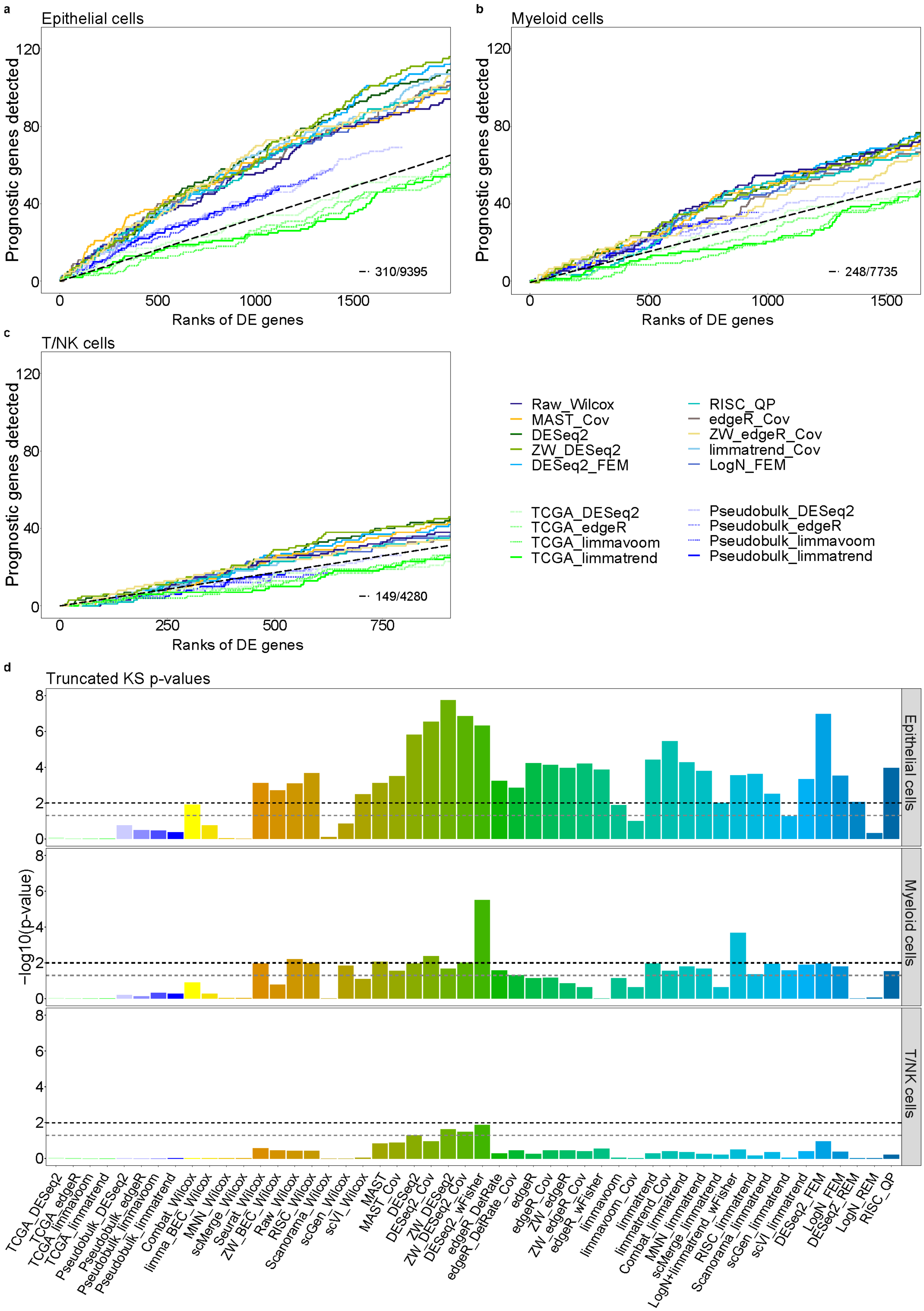
a
Epithelial cells
Prognostic genes detected
Ranks of DE genes
310/9395
b
Myeloid cells
Prognostic genes detected
Ranks of DE genes
248/7735
c
T/NK cells
Prognostic genes detected
Ranks of DE genes
149/4280
Raw_Wilcox
MAST_Cov
DESeq2
ZW_DESeq2
DESeq2_FEM
RISC_QP
edgeR_Cov
ZW_edgeR_Cov
limmatrend_Cov
LogN_FEM
TCGA_DESeq2
TCGA_edgeR
TCGA_limmavoom
TCGA_limmatrend
Pseudobulk_DESeq2
Pseudobulk_edgeR
Pseudobulk_limmavoom
Pseudobulk_limmatrend
d
Truncated KS p-values
−log10(p-value)
Epithelial cells
Myeloid cells
T/NK cells
TCGA_DESeq2
TCGA_edgeR
TCGA_limmavoom
TCGA_limmatrend
Pseudobulk_DESeq2
Pseudobulk_edgeR
Pseudobulk_limmavoom
Pseudobulk_limmatrend
Combat_Wilcox
limma_BEC_Wilcox
MNN_Wilcox
scMerge_Wilcox
Seurat_Wilcox
ZW_BEC_Wilcox
Raw_Wilcox
RISC_Wilcox
Scanorama_Wilcox
scGen_Wilcox
scVI_Wilcox
MAST
MAST_Cov
DESeq2
DESeq2_Cov
ZW_DESeq2
ZW_DESeq2_Cov
DESeq2_wFisher
edgeR_DetRate
edgeR_DetRate_Cov
edgeR
edgeR_Cov
ZW_edgeR
ZW_edgeR_Cov
edgeR_wFisher
limmavoom
limmavoom_Cov
limmatrend
limmatrend_Cov
Combat_limmatrend
MNN_limmatrend
scMerge_limmatrend
LogN+limmatrend_wFisher
RISC_limmatrend
Scanorama_limmatrend
scGen_limmatrend
scVI_limmatrend
DESeq2_FEM
LogN_FEM
DESeq2_REM
LogN_REM
RISC_QP

Figure S13. **Detection of prognostic genes compared between differential expression (DE) analyses of scRNA-seq, TCGA lung adenocarcinoma (LUAD) RNA-seq and pseudobulk data**. Cumulative counts of prognostic genes detected within top 20% DE gene ranks are shown for three cell types of LUAD: **a** epithelial cells, **b** myeloid cells and **c** T/NK cells. X-axis represents the gene ranks in each DE workflow (up to top 20%). Y-axis represents the cumulative counts of prognostic genes captured within top-*k* gene ranks by each DE workflow. The black-dashed slopes represent the expected cumulative counts of prognostic genes for random gene ranks. Ten and four methods are selected for DE analyses of scRNA-seq and bulk/pseudobulk data, respectively. **d** *p*-values of truncated Komogorov-Smirnov (KS) test for DE analyses of scRNA-seq and bulk/pseudobulk data are shown for the three cell types. Black and gray dashes represent the two significance cutoffs *p*-values = 0.01 and 0.05, respectively. n = 7728, 17348 and 15293 cells were analyzed for epithelial, myeloid and T/NK cells data, respectively. 9395, 7735 and 4280 genes commonly found in both scRNA-seq and TCGA LUAD RNA-seq data were analyzed for epithelial, myeloid and T/NK cells, respectively.

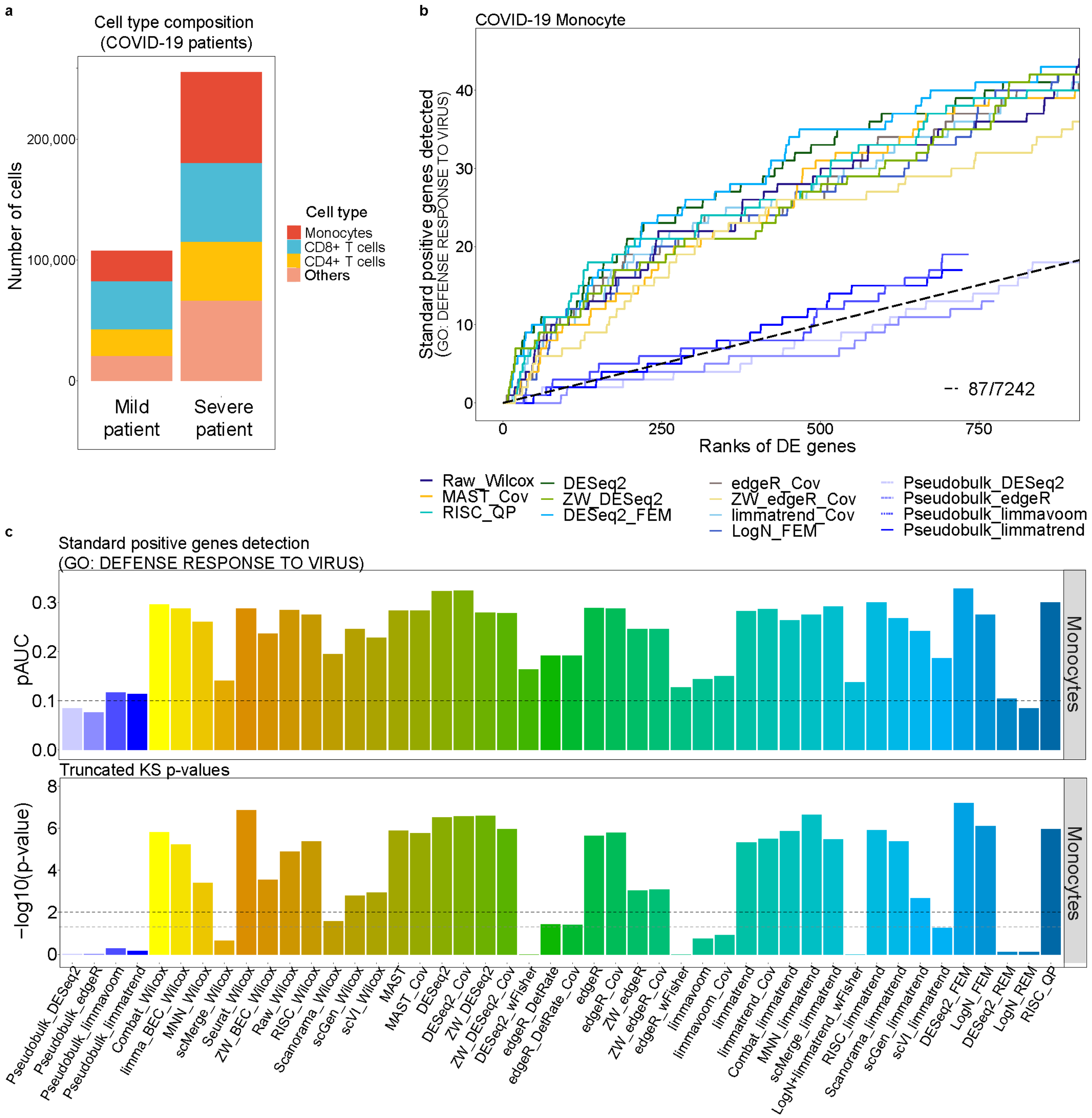

Figure S14. **Differential expression (DE) analysis on COVID-19 monocytes scRNA-seq data**. **Detection of virus defense genes in Gene Ontology (GO) is compared between DE analyses of scRNA-seq and pseudobulk data. a** Composition of cell types in mild and severe COVID-19 patient samples. **b** Cumulative counts of virus defense genes up to top 20% DE gene ranks are shown for monocytes. X-axis represents the gene ranks in each DE analysis. Y-axis represents the cumulative counts of virus defense genes captured within top-*k* gene ranks by each DE method. The black-dashed slope represents the expected cumulative number of virus defense genes for random gene ranks. Ten and four DE methods are selected for analyses of scRNA-seq and pseudobulk data, respectively. **c** Partial areas under the curve (pAUCs) and *p*-values of truncated Komogorov-Smirnov (KS) test for DE analyses of scRNA-seq data are shown. For pAUC, black-dashed line represents pAUC estimated from the expected numbers of standard positive genes (virus defense genes) for random gene ranks. For *p*-values, black and gray dashes represent the two significance cutoffs *p*-value = 0.01 and 0.05, respectively. 7242 genes and 100361 cells were used in COVID-19 monocyte data analysis.

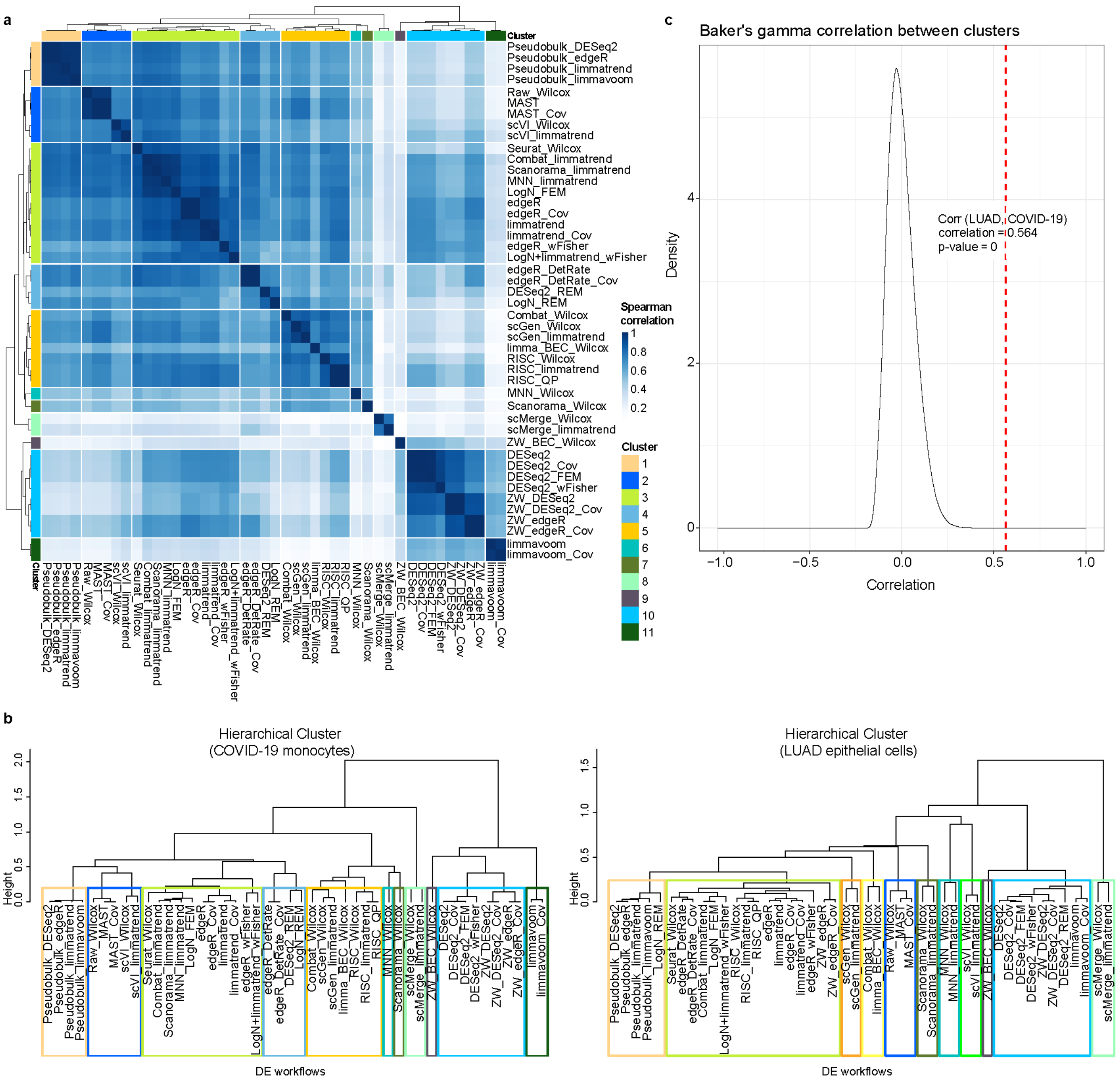

Figure S15. **Hierarchical clustering of differential expression (DE) analysis results based on spearman rank correlation. a** Clustering heatmap of 46 DE workflows for COVID-19 monocyte data. **b** Dendrograms of hierarchical clustering for COVID-19 monocyte and lung adenocarcinoma (LUAD) epithelial cell data. **c** Baker's gamma correlation between the clustering results for LUAD epithelial cell and COVID-19 monocyte data (Red dash). The density function of correlation was estimated from one million permutations of the clustering nodes. Ranks of 7242 and 10278 DE genes from COVID-19 monocyte and LUAD epithelial cells data were used for clustering DE workflows, respectively.

Supplementary Tables

Table S1. Main features of simulation data used in this study. The table shows the numbers of cells, genes (filtered) and differentially expressed genes (DEGs), as well as average depth, maximum read count and sparsity (zero rate) in each batch.

Datasets	Pancreas	T-cell	B-cell	Splatter (depth-77)	Splatter (depth-4)	Splatter (depth-10)
Batch1-Cells	562	358	184	300	300	300
Batch2-Cells	378	266	500	750	750	750
Genes (filtered)	7168	3059	2609	1997	2963	2745
DEGs	1434	612	522	343	557	539
Batch 1						
Avg. depth	2.66	1.99	1.78	78.04	4.11	10.51
Max count	2173	168	49	2840	286	562
Sparsity	0.75	0.83	0.83	0.76	0.72	0.68
Batch 2						
Avg. depth	2.76	270.60	282.40	76.60	4.06	10.33
Max count	2422	58702	68790	1649	184	654
Sparsity	0.78	0.60	0.78	0.76	0.73	0.68

Reference

1 Duò, A., Robinson, M. D. & Soneson, C. A systematic performance evaluation of clustering methods for single-cell RNA-seq data. *F1000Res* **7**, 1141 (2018). https://doi.org:10.12688/f1000research.15666.3

2 Sergina, N. V. & Moasser, M. M. The HER family and cancer: emerging molecular mechanisms and therapeutic targets. *Trends Mol Med* **13**, 527-534 (2007). https://doi.org:10.1016/j.molmed.2007.10.002

3 Yang, H., Liang, S. Q., Schmid, R. A. & Peng, R. W. New Horizons in KRAS-Mutant Lung Cancer: Dawn After Darkness. *Front Oncol* **9**, 953 (2019). https://doi.org:10.3389/fonc.2019.00953

4 van Roy, F. & Berx, G. The cell-cell adhesion molecule E-cadherin. *Cell Mol Life Sci* **65**, 3756-3788 (2008). https://doi.org:10.1007/s00018-008-8281-1

5 Bremnes, R. M., Veve, R., Hirsch, F. R. & Franklin, W. A. The E-cadherin cell-cell adhesion complex and lung cancer invasion, metastasis, and prognosis. *Lung Cancer* **36**, 115-124 (2002). https://doi.org:10.1016/s0169-5002(01)00471-8

6 Johnson, W. E., Li, C. & Rabinovic, A. Adjusting batch effects in microarray expression data using empirical Bayes methods. *Biostatistics* **8**, 118-127 (2006). https://doi.org:10.1093/biostatistics/kxj037

7 Haghverdi, L., Lun, A. T. L., Morgan, M. D. & Marioni, J. C. Batch effects in single-cell RNA-sequencing data are corrected by matching mutual nearest neighbors. *Nat Biotechnol* **36**, 421-427 (2018). https://doi.org:10.1038/nbt.4091

8 Lin, Y. *et al.* scMerge leverages factor analysis, stable expression, and pseudoreplication to merge multiple single-cell RNA-seq datasets. *PNAS* **116**, 9775-9784 (2019). https://doi.org:10.1073/pnas.1820006116

9 Ritchie, M. E. *et al.* limma powers differential expression analyses for RNA-sequencing and microarray studies. *Nucleic Acids Res* **43**, e47 (2015). https://doi.org:10.1093/nar/gkv007

10 Stuart, T. *et al.* Comprehensive Integration of Single-Cell Data. *Cell* **177**, 1888-1902.e1821 (2019). https://doi.org:10.1016/j.cell.2019.05.031

11 Wilcoxon, F. Individual comparisons by ranking methods. *Biom Bull* **1**, 80–83 (1945).

12 Finak, G. *et al.* MAST: a flexible statistical framework for assessing transcriptional changes and characterizing heterogeneity in single-cell RNA sequencing data. *Genome Biol* **16**, 278 (2015). https://doi.org:10.1186/s13059-015-0844-5
13 Love, M. I., Huber, W. & Anders, S. Moderated estimation of fold change and dispersion for RNA-seq data with DESeq2. *Genome Biol* **15**, 550 (2014). https://doi.org:10.1186/s13059-014-0550-8
14 Zhu, A., Ibrahim, J. G. & Love, M. I. Heavy-tailed prior distributions for sequence count data: removing the noise and preserving large differences. *Bioinformatics* **35**, 2084-2092 (2018). https://doi.org:10.1093/bioinformatics/bty895
15 Robinson, M. D., McCarthy, D. J. & Smyth, G. K. edgeR: a Bioconductor package for differential expression analysis of digital gene expression data. *Bioinformatics* **26**, 139-140 (2010). https://doi.org:10.1093/bioinformatics/btp616
16 Soneson, C. & Robinson, M. D. Bias, robustness and scalability in single-cell differential expression analysis. *Nat Methods* **15**, 255-261 (2018). https://doi.org:10.1038/nmeth.4612
17 Risso, D., Perraudeau, F., Gribkova, S., Dudoit, S. & Vert, J.-P. A general and flexible method for signal extraction from single-cell RNA-seq data. *Nat Commun* **9**, 284 (2018). https://doi.org:10.1038/s41467-017-02554-5
18 Squair, J. W. *et al.* Confronting false discoveries in single-cell differential expression. *Nat Commun* **12**, 5692 (2021). https://doi.org:10.1038/s41467-021-25960-2
19 Choi, J. K., Yu, U., Kim, S. & Yoo, O. J. Combining multiple microarray studies and modeling interstudy variation. *Bioinformatics* **19 Suppl 1**, i84-90 (2003). https://doi.org:10.1093/bioinformatics/btg1010
20 Wang, X. *et al.* An R package suite for microarray meta-analysis in quality control, differentially expressed gene analysis and pathway enrichment detection. *Bioinformatics* **28**, 2534-2536 (2012). https://doi.org:10.1093/bioinformatics/bts485
21 Fisher, R. A. *Statistical Methods for Research Workers*. 4th edn, (Oliver and Boyd, 1932).
22 Yoon, S., Baik, B., Park, T. & Nam, D. Powerful p-value combination methods to detect incomplete association. *Sci Rep* **11**, 6980 (2021). https://doi.org:10.1038/s41598-021-86465-y
23 Gayoso, A. *et al.* A Python library for probabilistic analysis of single-cell omics data. *Nat. Biotechnol.* **40**, 163-166 (2022). https://doi.org:10.1038/s41587-021-01206-w
24 Lotfollahi, M., Wolf, F. A. & Theis, F. J. scGen predicts single-cell perturbation responses. *Nature Methods* **16**, 715-721 (2019). https://doi.org:10.1038/s41592-019-0494-8
25 Hie, B., Bryson, B. & Berger, B. Efficient integration of heterogeneous single-cell transcriptomes using Scanorama. *Nat. Biotechnol.* **37**, 685-691 (2019). https://doi.org:10.1038/s41587-019-0113-3
26 Liu, Y., Wang, T. & Zheng, D. RISC: robust integration of single-cell RNA-seq datasets with different extents of cell cluster overlap. *bioRxiv*, 483297 (2018). https://doi.org:10.1101/483297
