# Supplementary material for: Benchmarking integration of single-cell differential expression
Source: Nat Commun. 2023 Mar 21;14:1570. doi: 10.1038/s41467-023-37126-3 (PMC10030080; doi:10.1038/s41467-023-37126-3)
Supplement: Supplementary file 3 — Description of Additional Supplementary Files [file 41467_2023_37126_MOESM3_ESM.pdf]

Description of Additional Supplementary Files

Supplementary Data 1: Top 20% Differential expression (DE) genes obtained from analysis of epithelial cell data using four scRNA-seq DE workflows and analysis of TCGA LUAD data using four bulk RNA-seq DE methods. Gene names, standard positive LUAD genes, adjusted p-values and logFC values for each gene were shown. Genes were sorted by their adjusted DE p-values.

Supplementary Data 2. The numbers of standard positive pathways in 16 cancer-related categories detected from GSEA of DE results for lung adenocarcinoma. Significant standard positive pathways (FDR < 0.25) within their ranks < 50 were counted for each category.

Supplementary Data 3. GSEA results for four scRNA-seq differential expression (DE) workflows applied to LUAD epithelial cell data and those for DE analysis of TCGA data. Pathways, corresponding standard positive categories, adjusted p-values, and enrichment scores from each method were described. The results were sorted by adjusted p-values for each DE method.
