# Supplementary material for: Benchmarking integration of single-cell differential expression
Source: Nat Commun. 2023 Mar 21;14:1570. doi: 10.1038/s41467-023-37126-3 (PMC10030080; doi:10.1038/s41467-023-37126-3)
Supplement: Supplementary file 7 — Reporting Summary [file 41467_2023_37126_MOESM7_ESM.pdf]

nature portfolio

Corresponding author(s): Dougu Nam, NCOMMS-22-22035C

Last updated by author(s): 2023-2-28

Reporting Summary

Nature Portfolio wishes to improve the reproducibility of the work that we publish. This form provides structure for consistency and transparency in reporting. For further information on Nature Portfolio policies, see our Editorial Policies and the Editorial Policy Checklist.

Statistics

For all statistical analyses, confirm that the following items are present in the figure legend, table legend, main text, or Methods section.

n/a	Confirmed	
☐	☒	The exact sample size (*n*) for each experimental group/condition, given as a discrete number and unit of measurement
☐	☒	A statement on whether measurements were taken from distinct samples or whether the same sample was measured repeatedly
☐	☒	The statistical test(s) used AND whether they are one- or two-sided<br>*Only common tests should be described solely by name; describe more complex techniques in the Methods section.*
☐	☒	A description of all covariates tested
☐	☒	A description of any assumptions or corrections, such as tests of normality and adjustment for multiple comparisons
☒	☐	A full description of the statistical parameters including central tendency (e.g. means) or other basic estimates (e.g. regression coefficient) AND variation (e.g. standard deviation) or associated estimates of uncertainty (e.g. confidence intervals)
☐	☒	For null hypothesis testing, the test statistic (e.g. *F*, *t*, *r*) with confidence intervals, effect sizes, degrees of freedom and *P* value noted<br>*Give P values as exact values whenever suitable.*
☒	☐	For Bayesian analysis, information on the choice of priors and Markov chain Monte Carlo settings
☒	☐	For hierarchical and complex designs, identification of the appropriate level for tests and full reporting of outcomes
☐	☒	Estimates of effect sizes (e.g. Cohen's *d*, Pearson's *r*), indicating how they were calculated

Our web collection on statistics for biologists contains articles on many of the points above.

Software and code

Policy information about availability of computer code

Data collection	An R package, "GEOquery", version 2.66.0 was used to collect microarray expression data from GEO database.
Data analysis	Python: anndata 0.8.0, helpers 0.2.0, matplotlib 3.5.3, numpy 1.23.1, pandas 1.4.4, scanorama 1.7.2, scanpy 1.9.1, scgen 2.1.0, scipy 1.9.1, scvi 0.6.8, seaborn 0.12.1, torch 1.12.1<br>R: Splatter 1.18.2, sva 3.38.0, batchelor 1.6.3, scMerge 1.6.0, limma 3.46.0, Seurat 4.0.2, Seurat Data 3.0.2, MAST 1.16.0, DESeq2 1.30.1, edgeR 3.32.1, ZINB-WaVE 1.12.0, fgsea 1.24.0, reshape2 1.4.4, ggplot2 3.4.1, magrittr 2.0.3, dplyr 1.1.0, cluster 2.1.4, factoextra 1.0.7, dendextend 1.16.0, GEOquery 2.66.0, biomaRt 2.54.0, survival 3.5-3, readr 2.1.4, stringr 1.5.0, Matrix 1.5-3<br><br>All codes for simulation and real data anlyses are provided in the public repository github (https://github.com/noobCoding/Benchmarking-integration-of-differential-expression/)

For manuscripts utilizing custom algorithms or software that are central to the research but not yet described in published literature, software must be made available to editors and reviewers. We strongly encourage code deposition in a community repository (e.g. GitHub). See the Nature Portfolio guidelines for submitting code & software for further information.

Data

Policy information about availability of data

All manuscripts must include a data availability statement. This statement should provide the following information, where applicable:

- Accession codes, unique identifiers, or web links for publicly available datasets
- A description of any restrictions on data availability
- For clinical datasets or third party data, please ensure that the statement adheres to our policy

This study includes reanalysis of published data as described in Methods and Data availability sections. The downloading sites and coresponding accession codes are as follows:
Single-cell lung adenocarcinoma data
GSE131907: [https://www.ncbi.nlm.nih.gov/geo/query/acc.cgi?acc=GSE131907]
Single-cell COVID-19 data
GSE131907: [https://www.ncbi.nlm.nih.gov/geo/query/acc.cgi?acc=GSE158055]
Microarray lung adenocarcinoma data
GSE29013: [https://www.ncbi.nlm.nih.gov/geo/query/acc.cgi?acc=GSE29013]
GSE30129: [https://www.ncbi.nlm.nih.gov/geo/query/acc.cgi?acc=GSE30129]
GSE31210: [https://www.ncbi.nlm.nih.gov/geo/query/acc.cgi?acc=GSE31210]
GSE37745: [https://www.ncbi.nlm.nih.gov/geo/query/acc.cgi?acc=GSE37745]
GSE43458: [https://www.ncbi.nlm.nih.gov/geo/query/acc.cgi?acc=GSE43458]
GSE50081: [https://www.ncbi.nlm.nih.gov/geo/query/acc.cgi?acc=GSE50081]

TCGA lung adenocarcinoma data are downloadable from the ucsc xenabrowser (https://xenabrowser.net/datapages/?dataset=TCGA-LUAD.htseq_counts.tsv&host=https%3A%2F%2Fgdc.xenahubs.net&removeHub=https%3A%2F%2Fxena.treehouse.gi.ucsc.edu%3A443).

Pathway data are downloadable from Enrichr Gene-set Library.
WikiPathway_2021_Human:[https://maayanlab.cloud/Enrichr/geneSetLibrary?mode=text&libraryName=WikiPathway_2021_Human]
GO_Biological_Process_2021: [https://maayanlab.cloud/Enrichr/geneSetLibrary?mode=text&libraryName=GO_Biological_Process_2021]
Known disease genes were downloaded from two public databases:
DisGeNET: [https://www.disgenet.org/]
CTD: [http://ctdbase.org/]

Human research participants

Policy information about studies involving human research participants and Sex and Gender in Research.

Reporting on sex and gender	N/A
Population characteristics	N/A
Recruitment	N/A
Ethics oversight	N/A

Note that full information on the approval of the study protocol must also be provided in the manuscript.

Field-specific reporting

Please select the one below that is the best fit for your research. If you are not sure, read the appropriate sections before making your selection.

☒ Life sciences ☐ Behavioural & social sciences ☐ Ecological, evolutionary & environmental sciences

For a reference copy of the document with all sections, see nature.com/documents/nr-reporting-summary-flat.pdf

Life sciences study design

All studies must disclose on these points even when the disclosure is negative.

Sample size	No statistical tests were performed for sample size calculation.<br>The number of samples used are described in the legend of each figure.<br>scRNA-seq Lung adenocarcinoma data<br>-Epithelial cells (n=7728)<br>-Myeloid cells (n=17348)<br>-T/NK cells (n=15293)

	scRNA-seq COVID-19 data<br>-Monocytes (n=100361)<br><br>TCGA Lung adenocarcinoma data (n=585)<br><br>Lung adenocarcinoma microarray expression data<br>-GSE31210 (n=241)<br>-GSE43458 (n=110)<br><br>For false positive/false discovery test,<br>-normal epithelial cells (n=2331)<br>-model-based simulation (n=2400)<br><br>For model-based simulations<br>-2-batch data (n=1000, 1050)<br>-7-batch data (n=2400)<br><br>For model-free simulations<br>-Pancreatic data (n=900)<br>-T-cell data (n=624)<br>-B-cell data (n=684)
Data exclusions	We excluded cells and genes from scRNA-seq count data as follows:<br>-mitochondrial genes <= 10 for LUAD data and <20 % for COVID-19 data.<br>-genes expressed in at least 5% of cells<br><br>In comparison of LUAD scRNA-seq data analysis and bulk sample expression data analysis, only genes common included in both datasets were used.<br><br>In all tests, genes expressed in less than 5% of cells were excluded.<br>In model-free simulations, all real marker genes were excluded.
Replication	Code for reproducing the results in this study can be found at https://github.com/noobCoding/Benchmarking-integration-of-differential-expression
Randomization	Randomization was not relevant as the study involved only re-analysis of published datasets.
Blinding	Blinding was not relevant as the study involved only re-analysis of published datasets.

Reporting for specific materials, systems and methods

We require information from authors about some types of materials, experimental systems and methods used in many studies. Here, indicate whether each material, system or method listed is relevant to your study. If you are not sure if a list item applies to your research, read the appropriate section before selecting a response.

Materials & experimental systems

n/a	Involved in the study
☒	☐ Antibodies
☒	☐ Eukaryotic cell lines
☒	☐ Palaeontology and archaeology
☒	☐ Animals and other organisms
☒	☐ Clinical data
☒	☐ Dual use research of concern

Methods

n/a	Involved in the study
☒	☐ ChIP-seq
☒	☐ Flow cytometry
☒	☐ MRI-based neuroimaging
